# H^+^-capacitor and ATP production in obligate alkaliphilic *Bacillaceae*: insights into cytochrome *c* and H^+^ transport mechanisms

**DOI:** 10.3389/fmicb.2025.1637315

**Published:** 2025-09-10

**Authors:** Isao Yumoto

**Affiliations:** Institute for Open and Transdisciplinary Research Initiatives (OTRI), The University of Osaka, Osaka, Japan

**Keywords:** membrane-bound cytochrome *c*, hydrogen-bonding network, H^+^ transfer amino acids, H^+^-capacitor, F_1_F_0_-ATP synthase

## Abstract

Alkaliphilic *Bacillaceae* strains likely utilize a limited number of free H^+^, producing ATP through an H^+^-based electrochemical membrane potential more efficiently than neutralophiles do. One possible mechanism responsible for this involves a structure that accumulates H^+^ through a hydrogen-bonding network formed by water molecules and the acidic, amido-, and hydroxyl- groups of amino acids located at the N-terminal site of membrane-bound cytochromes *c*, which are specifically found in obligate alkaliphiles. The segment of cytochromes *c* facilitates the formation of an H^+^-capacitor at the outer membrane surface. The H^+^-capacitor would produce an additional unbalanced vertical force to drive F_1_F_0_-ATP synthase via H^+^ concentrations and electrical charges across the membrane. Accumulated H^+^ ions are transferred from cytochrome *c* to the H^+^ influx gate of the *a*-subunit of F_1_F_0_-ATP synthase. However, the relative abundance of protonable basic amino acids at this site is low, suggesting that H^+^ transfer occurs via a membrane-bound protein containing the DUF2759 domain. This protein exposes basic amino acids that outnumber the deprotonatable acidic amino acids, effectively recruiting H^+^ from cytochrome *c* near the H^+^ influx gate of F_1_F_0_-ATP synthase. The disparity in abundance between acidic and basic amino acids within the H^+^ carrier segment may play a crucial role in determining H^+^ transfer efficiency. In alkaliphiles, significant gaps in H^+^ release or acceptance exist between the outer membrane and the intracellular side of F_1_F_0_-ATP synthase. This indicates that the hydrophilic segments involved in H^+^ transfer are specifically designed to enhance the performance of F_1_F_0_-ATP synthase. This hypothetical mechanism for the effective transportation of accumulated H^+^ to the N-terminal region of the cytochrome *c* amino acid sequence is essential for ATP production in obligate alkaliphilic *Bacillaceae*. The unique bioenergetic configuration of these alkaliphiles is evident in their high maximum ATP production rates. Maximizing the activity of F_1_F_0_-ATP synthase can be achieved through efficient H^+^ transport and a high transmembrane electrical potential (ΔΨ), particularly in environments where H^+^ availability is limited.

## 1 Introduction

Microorganisms thrive in various environments on Earth. This is due to their wide distribution and high adaptability to natural environments. Thus, studying the physiology of microorganisms is key to understanding the relationship between biological systems and their environments. Microorganisms that can adapt to extreme environments, such as low or high temperatures and pH levels, UV radiation, and high salinity, are called extremophiles (Satyanarayana et al., 2005; Rampelotto, 2013; Coker, 2023; Cowan et al., 2024). Studying organisms that live in extreme environments can improve our understanding of the hidden biological and biochemical mechanisms employed by microorganisms that live in normal environments (Goto et al., 2022). For instance, the physiological features observed during environmental adaptation in alkaliphiles may be present to a different extent in neutralophiles. We can gain a more comprehensive understanding of parameters sustaining biological systems by exploring these aspects in the microorganisms.

The mainstream view is that mutations favorable for environmental adaptation occur randomly and rarely at the same time, with adaptation then progressing through natural selection (Orr, 2005). However, microorganisms alter various cellular components and processes to adapt to alkaline environments. Given the phylogenetic proximity and synthesis of similar molecular features of proteins between facultative alkaliphiles and neutrophiles, the alkaliphilicity of facultative alkaliphiles may stem from physiological (e.g., transcriptional) gene regulation and transposable elements (Lebre and Cowan, 2020). Transposable elements are thought to have played an important role in evolution not only in facilitating horizontal gene transfer but also in gene rearrangements within genomes (Takami et al., 2001). Transposable elements in alkaliphilic bacteria may play diverse roles, such as enhancing genetic diversity, facilitating the transmission of resistance genes, regulating gene expression, and acquiring new functions for the corresponding environment (Siguier et al., 2014). This allows microorganisms to adapt and proliferate in alkaline environments. Over a long interaction period with environmental pH changes in a niche where the pH fluctuates between 7 and 10, facultative alkaliphiles with superior gene regulatory functions or post-translational modifications that facilitate adaptation to alkaline environments may have diverged.

Obligate alkaliphiles are often phylogenetically distant from that of neutralophiles, and they produce proteins with specialized molecular features for that support growth under high pH. These features may have originated from transposable elements and been retained through natural selection in persistently alkaline environments. Transposable elements and horizontal gene transfer are thought to contribute to the acquisition and diversification of alkaline-adaptive functions. These mechanisms may explain the coordinated changes in multiple cellular functions observed in alkaliphiles, contrasting with more gradual adaptation in neutralophiles.

Obligately alkaliphilic *Bacillaceae* within the phylum *Bacillota* (formerly *Firmicutes*) and class *Bacilli*, have been studied as model organisms for investigating bioenergetic adaptations to high-pH environments. On the other hand, other phylogenetically distinct obligately alkaliphilic bacteria also thrive in microbial communities adapted to high-pH serpentinizing systems by employing distinct physiological mechanisms to overcome the same energetic constraints. For example, *Alkaliphilus metalliredigens*, a strict anaerobe belonging to the phylum *Bacillota*, class *Clostridia*, could be thought to produce energy through metal reduction and potentially fermentative pathways (Ye et al., 2004). In contrast, aerobic hydrogen-oxidizing bacteria of the genus *Serpentinomonas*, classified within the phylum *Pseudomonadota* (formerly *Proteobacteria*), class *Gammaproteobacteria*, could be thought to generate ATP under alkaline conditions through hydrogen oxidation (Suzuki et al., 2014). In both cases, it is plausible that specific mechanisms exist to minimize H^+^ expenditure. These distinct metabolic strategies underscore the evolutionary adaptability of obligate alkaliphiles and emphasize the value of comparative studies across taxonomic lineages.

A key adaptation is the ability to maintain intracellular pH near neutrality, enabling survival in extreme alkaline conditions. Therefore, maintaining physiological mechanisms that regulate intracellular pH is essential for optimal metabolic activity within cells. *Caldalkalibacillus thermarum* strain TA2. A1, an alkaliphilic member of the family *Bacillaceae*, utilizes H^+^ ions to generate an electrochemical gradient that drives F₁F₀-ATP synthase (Dimroth and Cook, 2004). A Na^+^ potential could possibly be used across the membrane to drive F_1_F_0_-ATP synthase; in reality, alkaliphilic *Bacillaceae*, use H^+^ to produce ATP. However, anaerobic alkaliphilic *Alkaliphilus* spp. and the alkaliphilic, halotolerant cyanobacterium *Aphanothece halophytica*, use a Na^+^ potential across their membranes to drive ATP synthase (Hicks et al., 2010; Soontharapirakkul and Incharoensakdi, 2010). The reason why alkaliphilic *Bacillaceae* strains utilize H^+^ to drive F_1_F_0_-ATP synthase in H^+^-deficient environments remains unclear. However, using H^+^ for ATP production may be advantageous in some alkaliphiles (Banciu and Sorokin, 2013). For example, making more efficient use of scarce H^+^ would likely not require significant alterations to the physiological functions of the organism. Alkaliphilic *Bacillaceae* strains use the transmembrane Na^+^ potential (sodium motive force; SMF) for solute transport and flagellar rotation. The Na^+^/H^+^ antiporter, Mrp (Sha), plays a critical role in generating an SMF by translating the H^+^-based electrochemical potential generated by respiration into the transmembrane Na^+^ potential (Hamamoto et al., 1994; Krulwich et al., 2001). Through this translation, alkaliphilic *Bacillaceae* can dramatically reduce the frequency of H^+^ utilization. This key strategy uses limited H^+^ only to drive F_1_F_0_-ATP synthase. At the same time, the exchange of intracellular Na^+^ for extracellular H^+^ reduces the intracellular pH. Thus, the Na^+^/H^+^ antiporter plays a crucial role in conserving H^+^ for metabolism and in lowering intracellular pH levels (Krulwich et al., 2001).

In addition to utilizing ions, alkaliphiles protect themselves by displaying acidic substances on their cell surfaces. These negatively charged secondary cell walls repel OH^−^ under alkaline conditions, protecting cells from the environment. For instance, *Halalkalibacterium halodurans* (formerly *Bacillus halodurans*) C-125 exhibits teichuronopeptide and teichuronic acid on its cell surface as secondary cell walls (Aono and Horikoshi, 1983; Aono et al., 1993, 1995, 1999), whereas *Alkalihalobacillus pseudofirmus* OF4 exhibits cell surface layer (S-layer) protein A (SlaA) for this purpose (Gilmore et al., 2000). Although the substances displayed on the cell surface differ by species; they all play a crucial role in protecting cells from the harsh extracellular environment. Additionally, alkaliphilic *Bacillaceae* strains have been reported to produce acids to avoid direct interaction with harsh pH levels (Mano, 2020). Extracellular acid production mitigates the surrounding environment more effectively when bacterial cells exist as a group or live in a confined space. Thus, for alkaliphilic *Bacillaceae* strains, protection via the secondary cell wall and mitigation of the harsh environment around cells are important adaptation strategies to alkaline environments.

In addition to the previously described alkaline adaptation strategies, the membrane lipids that protect intracellular spaces differ between alkaliphilic *Bacillaceae* strains and their neutralophilic counterparts (e.g., *Bacillus subtilis*). These membrane lipid characteristics may play an important role in conferring the bioenergetic properties required for adaptation to alkaline environments. When alkaliphilic *Bacillaceae* strains use the respiratory chain to produce ATP by forming a H^+^-capacitor, the membrane lipid involved acts as a dielectric, providing insulation between the electrode plates of an electrical capacitor. Obligate alkaliphilic strains have 1.5 times more total membrane lipids than neutralophilic *B. subtilis* does (Clejan et al., 1986). The ratio of neutral to polar lipids is greater than ≥80% in obligate alkaliphiles, compared with the 33–54 and 43% in facultative alkaliphiles and *B. subtilis*, respectively. These results suggest that obligate alkaliphilic bacteria have fundamentally different physiological mechanisms than facultative alkaliphilic and neutralophilic bacteria do. Obligate and facultative alkaliphiles contain higher ratios of squalene, C_40_ isoprenoid, and negatively charged cardiolipin than *B. subtilis* does (Clejan et al., 1986). Cardiolipin may contribute to the negative charge of the membrane surface; however, the functions of squalene and C_40_ isoprenoid remain unknown.

Microorganisms require H^+^ to produce ATP. However, alkaliphiles have adapted to survive in conditions with one-thousandth of the H^+^ concentration found in neutralophilic conditions through use of the multiple physiologically supportive mechanisms described above. In addition to these features, alkaliphiles possess unique mechanisms for managing H^+^ through respiratory components. Due to the presence of a high transmembrane electrical potential (ΔΨ) across the membrane, the ability to retain H^+^ on the outer surface of the membrane (Yoshimune et al., 2010), and the H^+^-capacitor mechanism (Goto et al., 2022), alkaline environments are not harsh for alkaliphiles to survive in. The H^+^-capacitor mechanism is based on the Asn-rich N-terminal segment, which is specifically observed in the cytochrome *c* of obligately alkaliphilic *Evansella clarkii* (H^+^ carrier segment; Goto et al., 2022). The H^+^-capacitor function is enhanced under air-limited conditions by increased cytochrome *c* expression (Goto et al., 2022). This review summarizes and discusses the formation of an H^+^-capacitor and how H^+^ are transferred from the outer surface membrane to the intracellular membrane via F_1_F_0_-ATP synthase in alkaliphilic *Bacillaceae* for adapting to alkaline conditions.

## 2 Taxonomic background of alkaliphilic *Bacillaceae*

**Figure 1:**
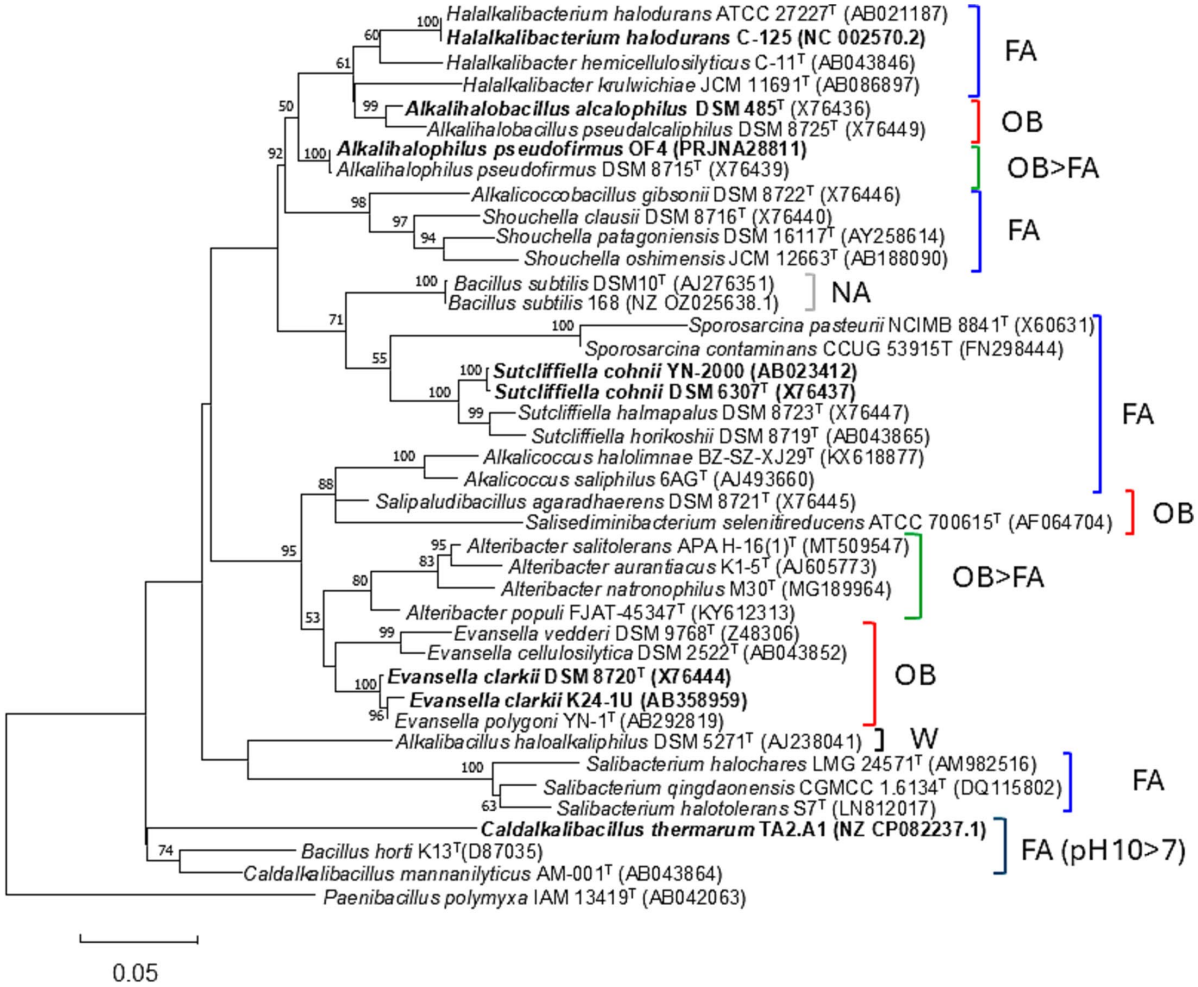
Maximum-Likelihood phylogenetic tree derived from the 16S rRNA gene sequences of obligate alkaliphilic *Evansella clarkii* and other related alkaliphilic and neutralophilic *Bacillaceae*. The evolutionary history was inferred by using the maximum-likelihood method and General Time Reversible model (Nei and Kumar, 2000). The tree with the highest log likelihood (−10998.89) is shown. The percentage of trees in which the associated taxa clustered together is shown alongside the branches. Initial tree(s) for the heuristic search were automatically obtained by applying the Neighbor-Join and BioNJ algorithms to a matrix of pairwise distances estimated using the Maximum Composite Likelihood approach and thereafter selecting the topology with a superior log likelihood value. A discrete Gamma distribution was used to model differences in evolutionary rate among sites (five categories [+G, parameter = 0.1885]). The rate variation model allowed for some sites to be evolutionarily invariable ([+I], 34.77% sites). The strains used in physiological studies for alkaline adaptation are indicated in bold text. The neutralophilic or alkaliphilic groups are categorized as follows: FA, facultative alkaliphiles; OB/FA, differ in obligate or facultative alkaliphiles depending on the strain; NA, neutralophilic; W, facultative alkaliphile that grows in a wider range than that of other facultative alkaliphiles; OB, obligate alkaliphiles; OB > FA, most of the species are obligate alkaliphiles, whereas a few strains are facultative alkaliphiles. FA (pH 10 > 7), facultative alkaliphiles, which grow much better in alkaline conditions than in neutralophilic conditions. The bootstrap values (>50%) based on 1,000 replications are shown in the branch node. *Paenibacillus polymyxa* IAM 13419^T^ was used as an outgroup. Bar, 0.05 substitutions per nucleotide position. Evolutionary analyses were conducted in MEGA11 (Tamura et al., 2021).

Since Vedder first isolated *Alkalihalobacillus alcalophilus* (formerly *Bacillus alcalophilus*) from the feces of healthy individuals in 1934, numerous alkaliphilic *Bacillaceae* strains have been discovered in various environments, including soil, animal manure, and ocean and lake water. Strains belonging to *A*. *alcalophilus* are obligate alkaliphiles, meaning that they cannot grow at a neutral pH. Facultative alkaliphiles, however, can grow at nearly the same intensity in both alkaline and neutral pH environments. The reason why many alkaliphiles have been isolated from normal environments remains unclear. Ammonia is naturally produced from the decomposition of proteins in animal urine. Ammonia could be released during the decomposition of proteins from dead plants and animals. Therefore, localized alkaline environments may be ubiquitous in nature, and alkaliphilic *Bacillaceae* may originate in these environments and later diffuse into normal environments. Facultative alkaliphiles are more frequently isolated than obligate alkaliphiles are from the normal soil samples used to isolate alkaliphilic *Bacillaceae* strains (Yumoto et al., 1997). A phylogenetic tree was constructed based on the 16S rRNA gene sequence (Figure 1). Each clade in the phylogeny exhibits characteristics of pH adaptation. For instance, the large clade comprising the genera *Halalkalibacterium*, *Alkalihalobacillus*, and *Shouchella* is facultatively alkaliphilic. In contrast, the clade containing the genera *Salisediminibacterium*, *Salipaludibacillus*, and *Evansella* is obligately alkaliphilic. However, the *Alkalibacillus haloalkaliphilus* strain isolated from these clades shows a wide growth pH range of 7.5–13. Thus, the strategies used for adapting to high pH levels are likely different in each class of alkaliphiles. Remarkably, we can infer that species exhibiting different growth characteristics for a pH range are not phylogenetically distant. The alkaliphilic species used thus far in bioenergetic studies to investigate alkaline adaptation mechanisms are as follows: *Halalkali*. *halodurans* (Hamamoto et al., 1994; Aono et al., 1996), *A. alcalophilus* (Lewis et al., 1980; Hoffmann and Dimroth, 1991), *Alkalihalophilus pseudofirmus* (formerly *Bacillus pseudofirmus*) (Lewis et al., 1980; Guffanti et al., 1986; Guffanti and Krulwich, 1994). *Sutcliffeiella cohnii* (formerly *Bacillus cohnii*) (Yumoto et al., 1991, 1993); *E. clarkii* (formerly *Bacillus clarkii*) (Ogami et al., 2009; Goto et al., 2022), and *Caldalkalibacillus thermarum* (formerly thermoalkaliphilic *Bacillus* sp.) (Meier et al., 2007; McMillan et al., 2009; Ferguson et al., 2016). Most of these strains are facultative alkaliphiles; only two, *A. alcalophilus* and *E. clarkii*, are obligate alkaliphiles. While each species employ their own strategy for adapting to high pH levels, each clade of alkaliphiles may contain common strategies to facilitate these adaptations.

## 3 Metal acquisition in alkaliphilic *Bacillaceae*

Alkaliphilic *Bacillaceae* face significant challenges in metal ion utilization for survival. Therefore, these organisms must possess efficient mechanisms for capturing and utilizing scarce metals. In most aqueous environments, ferric iron (Fe^3+^) tends to precipitate as insoluble Fe(OH)₃, significantly reducing its bioavailability above pH 6. This tendency becomes even worse in alkaline conditions. The concentration of iron available to microorganisms at pH 10 is estimated to be approximately 10^−23^ M (Drechsel and Jung, 1998), which is substantially lower than the 10^−18^ M typically found at pH 7 (McMillan et al., 2010). Limited iron availability impairs electron transfer efficiency, as respiratory chain components such as cytochromes and copper-dependent oxidases require precise metallation with Fe^2+^ and Cu^2+^ at the membrane interface, mediated by dedicated transport systems.

Although Fe^2+^ remains relatively soluble, it is highly susceptible to oxidation. In oxidative environments, Fe^2+^ is rapidly converted to Fe^3+^, further limiting its availability to microorganisms. If Fe^3+^ accumulates, it can lead to the production of toxic reactive oxygen species, such as oxygen free radicals. To circumvent this issue, alkaliphilic bacteria may adopt strategies to suppress oxidative conditions, thereby avoiding direct interactions with Fe^3+^. Alkaliphilic *Bacillaceae* strains encounter a shortage of Fe^2+^ and oxidative toxicity resulting from Fe^3+^.

To prevent iron deficiency and mitigate the toxicity of Fe^3+^, microorganisms secrete small molecules known as siderophores, that strongly bind to Fe^3+^ and facilitate its transport (Drechsel and Jung, 1998; Andrews et al., 2003). Approximately 500 types of siderophores have currently been reported, and in addition to iron ions, they can bind to other metal ions (e.g., Zn, Cu, Mo) (Ahmed and Holmström, 2014). Structural analyses of novel siderophores from alkaliphilic *Bacillaceae*, such as *Caldalkalibacillus thermarum*, have been documented (McMillan et al., 2010).

The genome of *A*. *pseudofirmus* OF4 reveals a six-gene cluster (asbABCDEF) responsible for synthesizing a catecholate siderophore (Janto et al., 2011). This siderophore is similar to Peribactin produced by the *Bacillus cereus* group. Adjacent to this gene cluster are four additional genes (*FhuGB* and *FhuRD*) that are predicted to encode an ABC-type siderophore transporter and its regulator, which is one of six ABC transporters anticipated to transport ferric siderophores. The genome also contains genes for two siderophore ferric reductases necessary for releasing iron from siderophore complexes through reduction. One reductase is located near a ferric dicitrate ABC uptake system, whereas the second reductase shows significant homology with proteins in *H*. *halodurans* and *Caldalkalibacillus thermarum*.

Another potential strategy used by alkaliphilic bacteria to prevent iron deficiency and mitigate oxidative conditions is the retention of metals by acidic compounds on the cell surface, such as polyglutamate capsules, teichuronopeptides, and teichuronic acid, which serve as secondary cell walls (Aono and Horikoshi, 1983; Aono et al., 1993, 1995, 1999), as well as S-layer protein A (SlaA) (Gilmore et al., 2000). Extracellular acidic polymers can accumulate deficient iron by adsorbing iron oxides, thereby reducing the direct toxic effects of Fe^3+^ on cells. These polymers may also alter the reactivity of Fe^3+^ by adjusting the surrounding pH and concentrations of other chemicals.

In some cases, alkaliphilic *Bacillaceae* species, such as *A*. *pseudofirmus*, exhibit metal ion-reducing abilities (Ma et al., 2012). Additionally, other *Bacillaceae* such as *S*. *cohnii* (Aino et al., 2018) and *Halalkalibacter hemicellulosilyticus* (Lopes et al., 2021) may also possess the ability to reduce indigo. The extracellular electron transfer capabilities of these organisms can reduce Fe^2+^, thereby detoxifying reactive oxygen species and enhancing the utilization of iron for their metabolic processes, particularly within their respiratory systems.

## 4 Bioenergetics in alkaliphilic *Bacillaceae*

According to Mitchell’s chemiosmotic theory (Mitchell, 1961), the H^+^ motive force (Δp) that drives F_1_F_0_-ATP synthase consists of the electrical potential difference (Δψ) across the membrane (ψ_in_ – ψ_out_) and the transmembrane pH gradient (ΔpH: pH_in_ – pH_out_).


Δp=Δψ–ZΔpH

$$Z=2.303\mathrm{RT}/\mathrm{zF}=\mathrm{ca}.59\;\mathrm{mV}\;(\mathrm{at}\;25^{{}^{\circ}}C)$$

where *R* = gas constant (8.315 J/K/mol), *T* = absolute temperature, and *F* = Faraday constant (96.485 kJ/mol/V). The constant holds for charge *z* = +1 |e| (i.e., H^+^).

Both the bulk-based bioenergetic parameters and growth rate of the facultatively alkaliphilic *A*. *pseudofirmus* OF4 on malate-containing medium were estimated at different culture pH levels under pH-controlled culture conditions (Sturr et al., 1994). The strain exhibited a specific growth rate and Δp potential of 1.10 h^−1^ and −26 mV, respectively, at pH 10.6. At pH 7.5, the specific growth rate and Δp were 0.77 h^−1^ and −140 mV, respectively. These results suggested that the bulk-based Δp did not reflect a real bioenergetic parameter. A similar observation was made in an experiment using right-side-out membrane vesicles (Guffanti and Krulwich, 1994). On ascorbate *plus* phenazine methosulfate energized membrane vesicles prepared from cells of the OF4 strain grown at pH 10.5, the Δp was measured at −36 mV (including bulk base ΔpH), and ATP production was 2.6 nmoles • mg protein^−1^ • 30 min^−1^. At pH 7.5, the energized membrane vesicles of cells generated a Δp and ATP production of −166 mV (including bulk base ΔpH) and 1.4 nmoles • mg protein^−1^ • 30 min^−1^, respectively. In addition, an artificially imposed K^+^ diffusion potential equivalent to the respiration-derived Δψ failed to synthesize ATP, whereas α-aminoisobutyric acid (AIB) uptake occurred. These results suggest that ATP production can be realized by the H^+^-based Δψ produced by the respiratory chain. If H^+^ are abundant in the extracellular space, ATP could be produced by the imposed K^+^-based diffusion potential. At the same time, alkaliphilic bacteria could transport the limited H^+^ translocated by the respiratory chain to the F_1_F_0_-ATPase in a unique way. Therefore, Guffanti and Krulwich (1994) hypothesized that H^+^ translocated across the membrane via respiratory complexes are directly transferred to the H^+^ influx gate of F_1_F_0_-ATP synthase.

This review discusses the H^+^-capacitor attributed to the Δψ and H^+^-transferable amino acids associated with water molecules. The H^+^-capacitor concept was first reported by Lee in 2012 (Lee, 2012, 2021). Based on the protonic capacitor concept, the ideal transmembrane electrostatically localized H^+^ (TELP) concentration [*H*_L_^+^]^0^ (mol/L = M) is given by the following equation:


$${\left[H_{L}^{+}\right]}^{0}=\frac{C}{S}\cdot \frac{\Delta \Psi }{l\cdot F}$$

where C/S is the specific membrane capacitance per unit surface area: *C* the capacitance in coul (electric charge)/V (voltage) (i.e., farads); *S* the area of the plates (m^2^); *l* the thickness (m) of the layer of transmembrane-electrostatically localized H^+^; and *F* the Faraday constant (96,486 coul/mol). In this review, configuration of the bioenergetic mechanisms of obligate alkaliphilic *E. clarkii* in alkaline conditions is mainly forced, and the system can be considered to show an enhanced version of the H^+^-capacitor compared with that of other organisms. Facultatively alkaliphilic *Bacillaceae* strains, while lacking the corresponding configuration of obligate alkaliphiles, can thrive in environments similar to those wherein obligate alkaliphiles grow. The TELP theory may explain the growth of facultative alkaliphilic strains in alkaline conditions.

## 5 Structure and function of cytochromes *c*

Cytochrome c is an electron-transfer protein that acts as a component of the respiratory chain by transferring electrons from the cytochrome *bc*_1_ complex (complex III) to cytochrome *c* oxidase. Compared with those in gram-positive bacteria, cytochromes *c* in gram-negative bacteria have been intensively studied (Yamanaka, 1992). One reason for this disparity is that cytochrome *c* in gram-negative bacteria is soluble, whereas in gram-positive bacteria, it is membrane-bound (Sone and Toh, 1994). Cytochromes *c* in gram-negative bacteria are thought to exist in the periplasmic space. However, cytochrome *c* in gram-positive bacteria is membrane-bound and has the same function as that of cytochrome *c* in gram-negative bacteria. As cytochrome *c* is membrane-bound in gram-positive bacteria, its movement is restricted; thus, fulfilling physiological functions that require accumulated cytochrome *c* is possible. Below, we discuss the molecular adaptation strategies of cytochromes *c* in alkaline environments by comparing them in neutralophilic, facultative, and obligate alkaliphilic bacteria.

### 5.1 Neutralophilic cytochromes *c*

**Figure 2:**
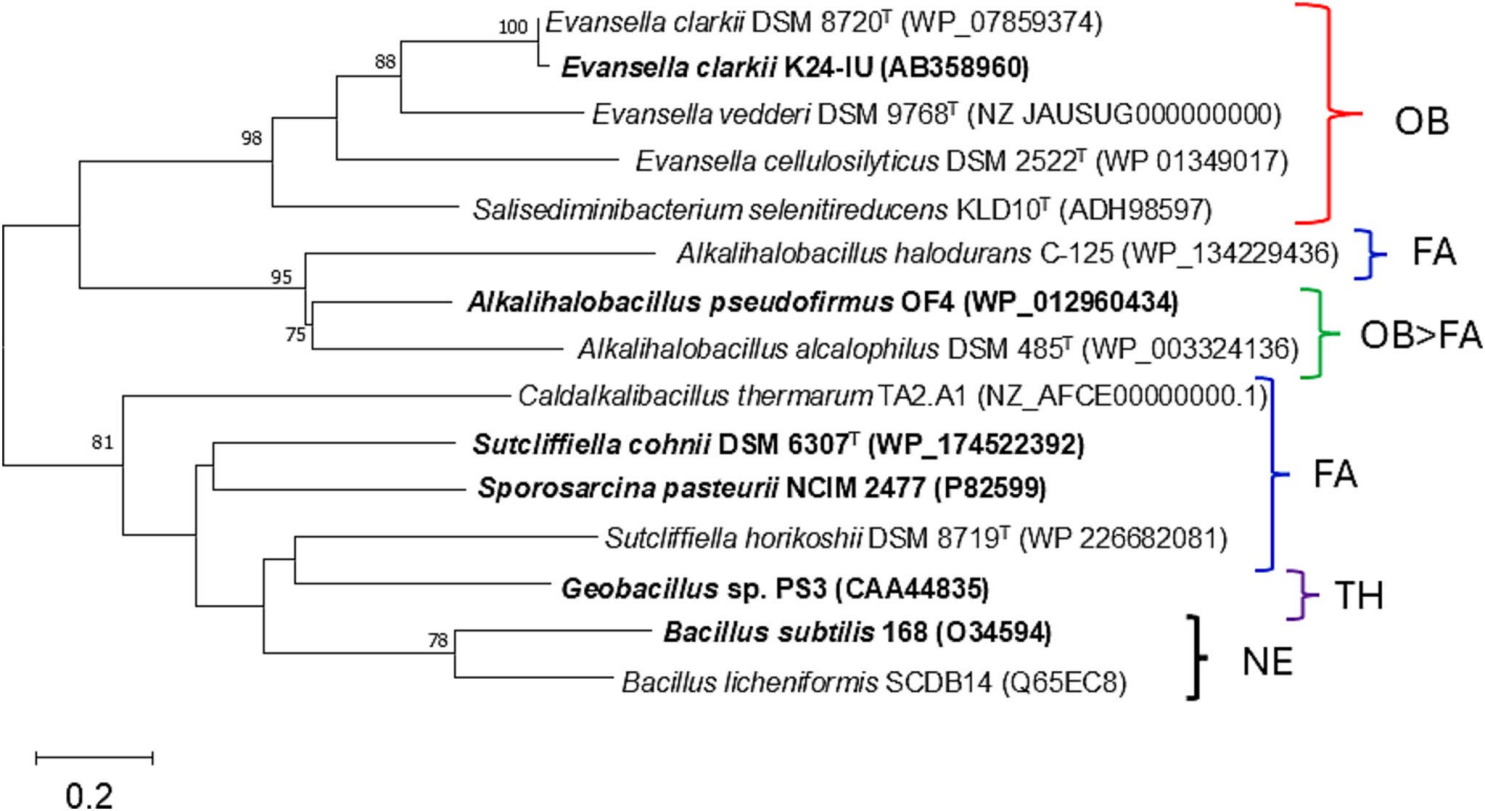
Maximum-likelihood phylogenetic tree of the membrane bound cytochromes *c* from *Evansella clarkii* and other alkaliphilic and neutralophilic *Bacillaceae*. Evolutionary history was inferred by using the Whelan and Goldman model (Whelan and Goldman, 2001). The tree with the highest log likelihood (−2807.82) is shown. The percentage of trees in which the associated taxa clustered together is shown alongside the branches. Initial tree(s) for the heuristic search were automatically obtained by applying the Neighbor-Join and BioNJ algorithms to a matrix of pairwise distances estimated using the JTT model and then selecting the topology with a superior log likelihood value. A discrete Gamma distribution was used to model differences in evolutionary rate among sites (five categories [+G, parameter = 4.1748]). The rate variation model allowed for some sites to be evolutionarily invariable ([+I], 8.25% sites). Strains or species for which protein studies have actually been conducted are indicated in bold. Bar, 0.2 substitutions per nucleotide position. Evolutionary analyses were conducted in MEGA11 (Tamura et al., 2021). The neutralophilic or alkalophilic strains were categorized as follows: OB, obligate alkaliphiles; FA, facultative alkaliphiles; OB > FA, most of the strains are obligate alkaliphiles, whereas a few are facultative alkaliphiles; NA, neutralophilic; TH, thermophile.

Few examples of studies on membrane-bound cytochrome *c* in *Bacillaceae* can be found; a typical example from neutralophilic bacteria is that of the *B. subtilis* cytochrome *c*. As *B. subtilis* exhibits strong protease activity, its cytochrome *c* has been reported as a soluble protein (Miki and Okunuki, 1969a,b). Two different membrane anchor types of cytochrome *c* (*c*-550 and *c*-551) from *B. subtilis* 168 were purified and characterized. Cytochrome *c*-551, which is equivalent to the cytochrome *c*-550 of *E. clarkii* discussed below, is composed of 112 amino acid residues, including a signal peptide sequence of 20 residues (Table 1; Bengtsson et al., 1999). The processed form, which is dissociated from the signal peptide, consists of 92 amino acids, including 18 acidic and 14 basic amino acids, and binds to the membrane via a diacylglyceryl moiety (the acyl chain length of which is C_15_) through its N-terminal cysteine. The redox potential of this cytochrome *c* was reported to be higher than +100 mV. The phylogenetic position of the amino acid sequence of *B. subtilis* cytochrome *c*-551 was found to be similar to that of *Bacillus licheniformis*, which is distantly related to the clade of obligate alkaliphilic strains (Figure 2). The other cytochrome *c*, cytochrome *c*-550, consists of approximately 120 amino acids, including a single α-helical transmembrane segment of approximately 30 hydrophobic amino acids, which serves as a membrane anchor (von Wachenfeldt and Hederstedt, 1993; David et al., 2000). These cytochromes *c* may likely have a function in electron transfer reactions between the *b*_6_*c* complex and cytochrome *c* oxidase (cytochrome *caa*_3_). An almost pure *b*_6_*c* complex associated with cytochromes *c*-550 and *c*-551 has been isolated previously (Picón Garrido et al., 2022). The estimated molecular masses of cytochromes *c*-550 and *c*-551 from *B. subtilis* were not reported via gel filtration. However, cytochrome *c*-551 from *Geobacillus* sp. PS3, which is equivalent to the cytochrome *c*-551 found in *B. subtilis*, was reported as a trimer via gel filtration (Table 1; Sone et al., 1989; Fujiwara et al., 1993). According to the amino acid sequence alignment of membrane-bound cytochromes *c*, *B. subtilis* cytochrome *c*-551, *Geobacillus* sp. PS3 cytochrome *c*-551, and *B. licheniformis* cytochrome *c* are classified as neutralophilic. The *B. subtilis* cytochrome *c*-551 is in the same clade as *B. licheniformis* cytochrome *c*, whereas *Geobacillus* sp. PS3 cytochrome *c*-551 is in the same clade with the facultatively alkaliphilic *Sutcliffiella horikoshii* cytochrome *c*. This may be reflected in the phylogenetic distance observed between the genera *Geobacillus* and *Bacillus*.

**Table 1:** Biochemical properties of membrane-bound cytochromes *c*.

Biochemical property	*Evansella clarkii* cytochrome *c*-550^a^	*Sutcliffiella cohnii* cytochrome *c*-553^b^	*Bacillus subtilis* cytochrome *c*-551^c^	*Geobacillus* sp. PS3 cytochrome *c*-551^d^
Category in environmental adaptation	Obligate alkaliphile*	Facultative alkaliphile	Neutralophile	Thermophile
Number of amino acids residues^e^	**101**	93	92	93
Molecular mass in SDS-PAGE (kDa)	**20, 17 (11.1)** ^i^	10.5 (9.7)	10 (11.0)	10.4 (10.4)
Molecular mass in gel-filtration (kDa)^f^	**40 (44.4)** ^j^ **, Tetramer**	37 (38.8), Tetramer	ND^g^	33 (31.2), Trimer
Anchor fatty acyl chain lengths (C_n_)	**15, 16, 17**	ND	15	15
Presence of N-terminal Asn^4^-Asn^27^ sequence^e^	**Yes**	No	No	No
Number of acidic and basic amino acid residues in the amino acid sequence corresponding to Asn^4^-Asn^37^ in *E. clarkii*^e^	**Acidic 10; Basic 0**	Acidic 4; Basic 0	Acidic 6; Basic 3	Acidic 2; Basic 1
Number of Asn, Ser, or Thr in the amino acid sequence corresponding to Asn^4^-Asn^37^ in *E. clarkii*^e^	**10**	4	5	7
Number of basic residues^e^	**2**	7	14	12
Midpoint redox potential (mV)^h^	**+83**	+87	˃+100	+225
p*I* (Isoelectric point)	4.1	3.9	3.8	4.0
Absorption maxima at				
Oxidized form (nm)	408	411	409	409
Reduced form (nm)	415, 521, 550	417, 524, 553	416, 522, 551	416, 522, 551

Each column represents data from a representative species of obligate alkaliphile, facultative alkaliphile, neutralophile, and thermophile, respectively.

*Properties highlighted in bold represent distinctive features of the membrane-bound cytochrome c from the obligate alkaliphilic bacterium *Evansella clarkii*.

a Data obtained from Ogami et al. (2009) using *E. clarkii* K24-1U and the whole genome sequence of *E. clarkii* DSM 8720^T^ (NZ_MTIV00000000.1) were considered.

b Data obtained from Yumoto et al. (1991) using *S. cohnii* YN-2000 and the whole-genome sequence of *S. cohnii* DSM 8720^T^ (NZ_CP018866.1) were considered.

c Data obtained from Bengtsson et al. (1999).

d Data obtained from Sone et al. (1989) and Fujiwara et al. (1993).

e Processed proteins were considered.

f Values were subtracted from the molecular mass of Triton X-100.

g ND: No data.

h Values were estimated using redox titration at pH7.

i,j Theoretical value, including the heme, anchor, and N-terminal modifications.

### 5.2 Facultative alkaliphilic cytochromes *c*

**Figure 3:**
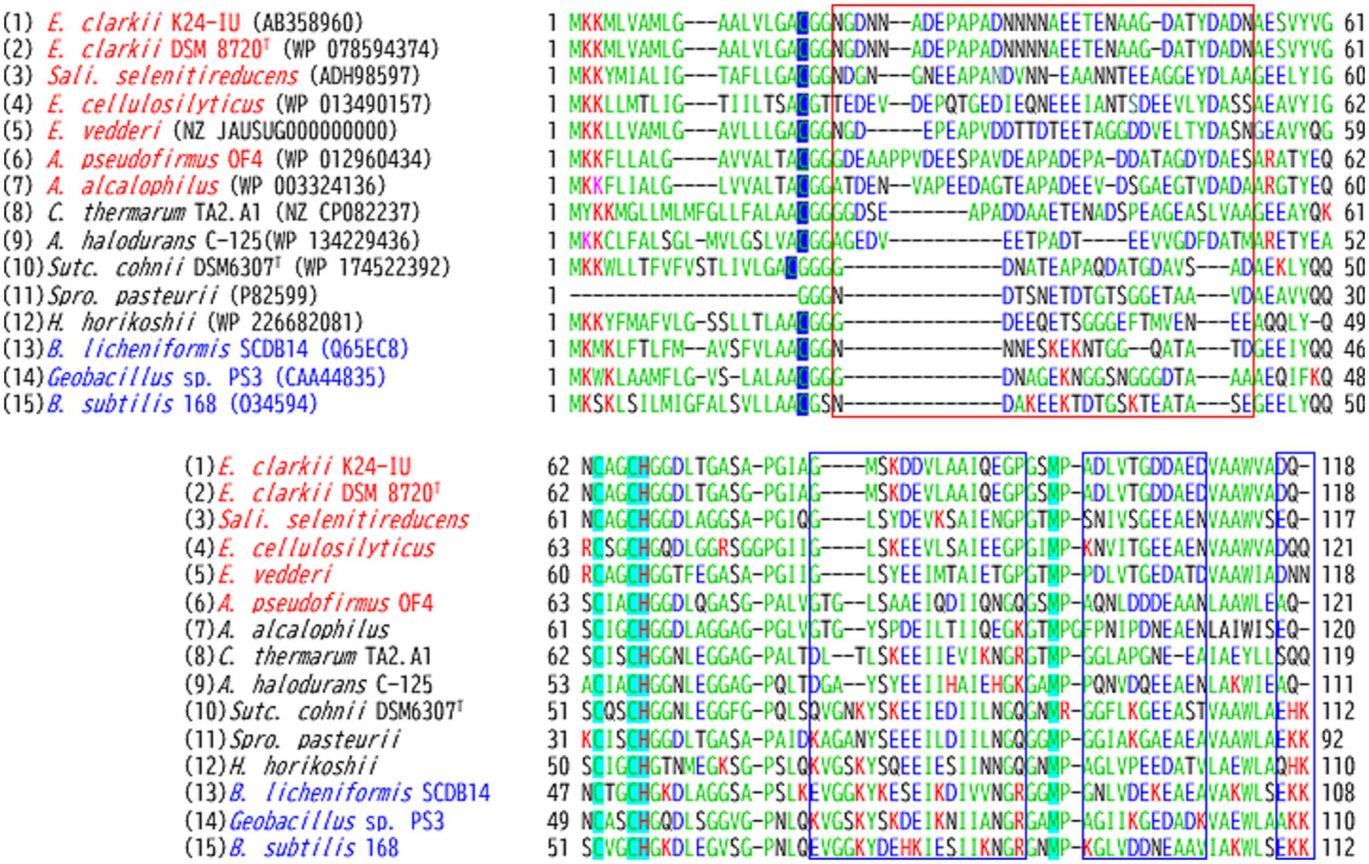
Amino acid sequence alignment of the membrane-bound cytochrome *c*-550 from *Evansella clarkii* and cytochromes *c* from other alkaliphilic and neutralophilic *Bacillaceae*. Obligate and facultative alkaliphilic strains are indicated by red and black, respectively, whereas neutralophilic strains are shown by blue. Asn (N)-rich segment: the Asn (N)^4^–Asn (N)^27^ sequence in the processed protein base (Asn [N]^21^–Asn [N]^44^ in the entire sequence base) in the *E. clarkii* cytochromes *c* and corresponding positions in other strains are indicated by a red box. The equivalent sequences were observed in obligate alkaliphilic strains ([1]–[7]). The N-terminal amino acid residue in processed cytochrome *c* (Cys [C]) is indicated by a blue marker. Amino acids representing the heme-binding site (C) and axical ligands (H and M) are indicated by a light blue marker. Acidic (D, E) and basic (H, K and R) residues are indicated by blue and red letters, respectively; amino acids representing amido (N, Q) or hydroxyl (S, T) group side-chains are indicated by black letters; and hydrophobic amino acids are shown in pale green. Sequences showing a trend from neutral to alkaliphilic (D + E) + (N + Q + T + S) > (H + K + R) are indicated by blue boxes. While the *A*. *pseudofirmus* OF4 is a facultative alkaliphile, most strains belonging to *A*. *pseudofirmus* are obligate alkaliphiles (Nielsen et al., 1995), and the cytochrome *c* sequence presented is classified as obligate alkaliphile.

The abundance of cytochrome *c* was found to be higher when grown at pH 10 than at pH 7 in reduced *minus* oxidized forms of the membrane of the facultatively alkaliphilic *S*. *cohnii* YN-2000 (Yumoto et al., 1991). The elution profile obtained after anion-exchange chromatography of the loaded crude membrane extract showed that an increase in cytochrome *c*-553 was the main reason for the observed increase in cytochrome *c* abundance. Therefore, cytochrome *c*-553 is likely responsible for adaptation to high pH levels in the strain YN-2000. Cytochrome *c*-553 has a molecular mass of 10.5 kDa (according to SDS-PAGE results), and the native molecular mass determined using gel filtration showed that it forms a tetramer. The phylogenetic position of the amino acid sequence of *S*. *cohnii* DSM 6307^T^ was similar to that of other facultative alkaliphilic species (Figure 2). It consists of 93 amino acids, including 14 acidic and 7 basic amino acids (Table 1; Figure 3). The redox potential was +87 mV in the pH range of 6–8. Cytochrome *c*-553 reacted strongly with its physiological electron acceptor, cytochrome *c* oxidase (cytochrome *aco*_3_), in the presence of poly-L-lysine. This suggests that positively charged molecules are necessary for effective binding between cytochromes *c*-553 and *aco*_3_ (Yumoto et al., 1993).

Both the biochemical properties and three-dimensional structure of cytochrome *c*-553 from *Sporosarcina pasteurii* (formerly *Bacillus pasteurii*) has been reported previously (Benini et al., 2000). Cytochrome *c*-553 consists of 92 amino acids in the mature protein, with an N-terminal cysteine. Therefore, it is thought to consist of 92 amino acids, including 13 acidic and 6 basic amino acids. This cytochrome also has a low redox potential of +47 mV (Benini et al., 1998, 2000). Its structural features indicate that most of the charges are localized to the opposite side of the heme edge suggesting that most of the charges are localized between the protein molecule and outer membrane surface. This may be related to the accumulation and transfer of H^+^ localized between the cytochrome *c* molecule and outer membrane surface. Analysis of physicochemical parameters showed that the solvent accessibility of heme *c* correlated with entropy (*ΔS*: disorder) in the relationship between enthalpy (*ΔH*: heat content) and *ΔS* in the Gibbs free energy equation (*ΔG* = *ΔH* – T*ΔS*; T: absolute temperature [in Kelvin]). This suggests a direct relationship between the main determinant of the electrochemical reduction potential (*ΔS*) and a structural parameter (heme *c* solvent exposure). The low midpoint redox potential of cytochrome *c*-553 from *S*. *pasteurii* could be explained by the reduction of *ΔS* via water-molecule extrusion, which increases solvent accessibility. The water molecules are produced from the hydration shell in the cytochrome molecule during heme *c* reduction.

In conclusion, the particular characteristics of cytochromes *c* from facultative alkaliphiles include a low redox potential (<+90 mV) and lower content of basic amino acids (<8 residues) in the mature protein. Phylogenetic positions of the cytochromes *c* of *S*. *cohnii* and *S*. *pasteurii* are relatively close to that of *B. subtilis* cytochrome *c*-551 when compared with those of the other alkaliphilic cytochromes *c*. As the *S*. *cohnii* cytochrome *c* is relatively similar to that of *B. subtilis*, a higher amount of cytochrome *c*-553 may be expressed for adaptation under high pH conditions. Cytochrome *c* expression intensity may depend on the requirement of an H^+^-retaining function during environmental adaptation.

### 5.3 Cytochromes *c* from obligate alkaliphiles

The first cytochrome *c* identified from an obligate alkaliphilic *Bacillaceae* strain was cytochrome *c*-552 from *A. pseudofirmus* RAB (formerly *Bacillus firmus* RAB) (Davidson et al., 1988), which was purified as a soluble protein and had a molecular mass of 16.5 kDa. This size is larger than expected for common *Bacillaceae* based on its corresponding gene sequence length. The mean redox potential of cytochrome *c*-552 is +66 mV at pH 7, which decreases at pH > 8.3 at a rate of −54 mV/pH. For example, its redox potential at pH 9.25 is +8 mV. According to resonance Raman spectroscopy, this change in midpoint redox potential is attributed to a switch in the sixth ligand of heme *c* from methionine to histidine when oxidized cytochrome *c* is reduced (Larsen et al., 1990). Although it is unclear whether the *A*. *pseudofirmus* RAB cytochrome *c*-552 is equivalent to the cytochrome *c* of *A*. *pseudofirmus* OF4, the methionine could not have been replaced by histidine in the fifth ligand of heme *c* based on the amino acid sequence (Figure 3). The *A*. *pseudofirmus* RAB cytochrome *c*-552 is autoxidizable and has been isolated in a soluble form; these characteristics suggest that this protein is partially digested by intrinsic proteases during purification.

As cytochromes *c* in *Bacillaceae* have been shown to be sensitive to intrinsic protease activity, an attempt was made to isolate obligate alkaliphilic *Bacillaceae* strains that exhibit weak protease activity. Consequently, cytochrome *c*-550 was purified and characterized from an *E. clarkii* K24-1 U (Ogami et al., 2009). The purified cytochrome *c*-550 exhibited dimeric (23 kDa) and tetrameric (40 kDa) states in blue native PAGE and gel filtration results, respectively. Cytochrome *c*-550 binds to fatty acids with carbon lengths of C_15_–C_17_ via glycerol-Cys_18_ as a membrane anchor. The molecular species of the fatty acid corresponded to its membrane fatty acid, suggesting that when cytochrome *c*-550 expression fluctuates with culture conditions, the fatty acid composition of the membrane is not strongly affected. The redox potential of cytochrome *c*-550 determined via redox titration was +83 mV (pH 7–9), whereas that determined through cyclic voltametric measurements was +7 mV (pH 6.8). A possible explanation for this difference is the reorientation of the protein backbone that likely occurs due to the effect that the electric field of the electrode has on cytochrome *c*-550. Electric field-induced redox potential shifts have been reported to occur due to Coulombic forces from an electrode (Rivas et al., 2005). The phylogenetic position of cytochromes *c* from obligate alkaliphilic bacteria is distant from that of neutralophilic *B. subtilis* cytochromes *c*-551 when compared with those from facultative alkaliphilic bacteria (Figure 2). This reflects that cytochromes *c* in obligate alkaliphiles have evolved to be even more specialized for functioning under alkaline environments. Two clades comprising the genera Evancella and Alkalihalobacillus are present in the cytochrome *c* phylogeny of obligate alkaliphiles. In this sense, cytochromes *c* of the *A*. *pseudofirmus* OF4 and *E. clarkii* K24-1 U belong to different clades.

In conclusion, the particular characteristics of cytochromes *c* from obligate alkaliphiles include a low redox potential (< +90 mV) and lower content of basic amino acids (< 3 residues) in the mature protein. Phylogenetic positions of the cytochromes *c* of *A*. *pseudofirmus* OF4 and *E. clarkii* are more distant from that of *B. subtilis* cytochrome *c*-551 when compared with those of the other alkaliphilic cytochromes *c*. Since the cytochrome *c* of *S. cohnii* is relatively similar to that of *B. subtilis*, compared to *A. pseudofirmus* OF4 and *E. clarkii*, a higher level of cytochrome *c* expression may contribute to adaptation under high-pH conditions. The intensity of cytochrome *c* expression may depend on the necessity of the H^+^-retaining function.

## 6 Comparison of amino acid sequences in cytochrome *c* and F_1_F_0_-ATP synthase

### 6.1 Cytochrome *c*

The amino acid sequence of Asn_4_-Asn_37_ in the mature protein base in *E. clarkii* cytochromes *c* is quite unique among obligate alkaliphiles compared with those of facultative and neutralophilic membrane-bound cytochromes *c* (Figure 3; Table 1). The Asn_4_-Asn_37_ sequence is located between the first N-terminal α-helix in the main body of the protein and the membrane anchor, based on the structural data of *S*. *pasteurii* (Benini et al., 2000). Between Asn_4_-Asn_37_, 10 acidic amino acids (glutamic acid [Glu (E)] or aspartic acid [Asp (D)]), nine amido group side-chains possessing asparagine [Asn (N)] and glutamine [Gln (Q)], and one hydroxyl group side-chain (possessing threonine [Thr (T)] and serine [Ser (S)]) can be found in *E. clarkii* cytochrome *c*. Glu (E) and Asp (D) are acidic amino acids with a H^+^-transferable carboxyl group on their side-chains (Voet et al., 2016). Asn (N), Gln (Q), Thr (T), and Ser (S) can act as hydrogen-bond acceptors and contribute to hydrogen-bond formation (Schoneich et al., 1994; Doukov et al., 2007; Vennelakanti et al., 2021). This structural feature contributes to the hydrogen-bond network in the space between the main body of the cytochrome *c*-550 molecule and outer membrane surface. The tetrameric structure of this cytochrome *c* can further enhance the advantages of its H^+^-transferring properties. The segment of the cytochromes *c* lead the formation of an H^+^-capacitor at the outer membrane surface. The H^+^-capacitor would produce an additional unbalanced vertical force to drive F_1_F_0_-ATP synthase via H^+^ concentrations and electrical charges across the membrane.

Alignment of the amino acid sequence of cytochromes *c* from obligate and facultative alkaliphilic and neutralophilic strains with *Bacillaceae* showed obvious difference among them (Figure 3). The pronounced differences between cytochromes *c* from obligate alkaliphilic strains and those of others are the paucity of basic amino acids and presence of long sequences between the main body of the cytochrome *c* molecule and three amino acids at the N-terminus. These membrane-bound cytochromes *c* can potentially regulate H^+^-transferability via the acid ratio and amino acid composition of the sequence adjacent to the N-terminal sequence. Acidic amino acids, such as Asp (D) and Glu (E), can deprotonate their carboxyl groups and release H^+^ under high pH, whereas basic amino acids, such as Lys (K) and Arg (R), can protonate their amino side-chains under low pH and thus become H^+^-receivers (Voet et al., 2016). Given that H^+^ tend to accumulate at the cell membrane surface, resulting in a local pH assumed to be below 8. Both arginine (Arg) and lysine (Lys) are expected to accept protons due to their high p*K*_a_ values (~12.5 and ~10.5, respectively). Therefore, the feature of the amino acid sequence of obligate alkaliphiles precedes H^+^-transfer to the H^+^-gate in F_1_F_0_-ATP synthase. However, the presence of amido groups on the side-chain possessing Asn (N), which act as proton carries, can accumulate H^+^ at the outer membrane surface. In addition to the N-terminal sequence, the C-terminal sequence and presence of two α-helices tend to decrease the frequency of basic amino acids and increase that of acidic amino acids.

### 6.2 Cytochrome *c* moiety in complexes III and IV

In addition to independent cytochromes *c*, such as the cytochrome *c*-550 of *E. clarkii*, cytochrome *c* components can exist in complexes, such as complexes III and IV. The subunit II of complex IV (cytochrome *c* oxidase in *S. cohnii* and *Shouchella lehensis*) has been reported to lack basic amino acids in its alkaliphilic form (Denda et al., 2001; Noor et al., 2014). Cytochromes *c* are attached to complexes III and IV in obligate alkaliphiles; a relatively higher acidic amino acid abundance *plus* the amino acids contributing to H^+^ transfer and a lower basic amino acid abundance could be present in areas exposed to the outer membrane surface (Supplementary Figures S1, S2). However, the pronounced trend in the abundance of Asn (N) observed in *E. clarkii* cytochrome *c*-550 and the other obligate alkaliphilic cytochromes *c* is not intense in the cytochrome *c* subunits in complexes III and IV.

In conclusion, all cytochromes *c*, including the cytochrome *c* moiety in complexes III and IV, are believed to cooperatively and efficiently transport the H^+^ that are pumped to the outer membrane surface via the respiratory chain to F_1_F_0_-ATP synthase and retain them on the outer membrane surface in conjunction in obligate alkaliphiles. However, independent cytochrome *c* contains more acidic and fewer basic amino acids than the cytochrome *c* moieties in complexes III and IV. Specificity of the additional N-terminal sequences may have higher efficiencies for H^+^ retention and transfer functions than those of the other moiety in the respiratory system. The location of the additional N-terminal sequence moiety in obligate alkaliphiles, which is found between the main cytochrome *c* molecule and membrane anchor moiety, may have a central role in coupling the redox properties of cytochrome *c*.

### 6.3 DUF2759 domain-containing protein and F_1_F_0_-ATP synthase: *a*- and *c*-subunits

**Figure 4:**
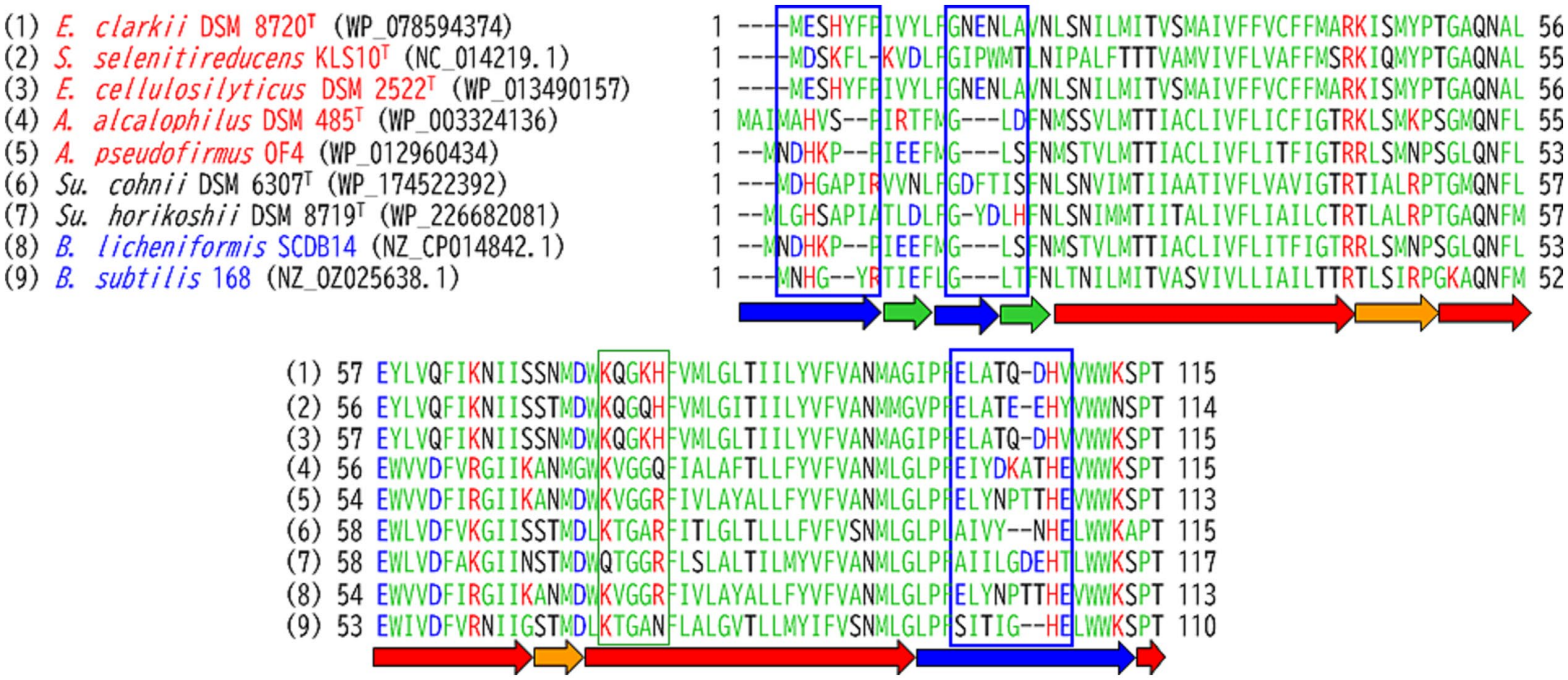
Amino acid sequence alignment of the *a*-subunit of F_1_F_0_-ATP synthase from *Evancella clarkii* DSM 8720^T^ and other alkaline and neutral hydrophilic *Bacillaceae*. Obligate and facultative alkaliphilic strains are indicated by red and black, respectively, and neutralophilic strains shown by blue. Green and red arrows indicate β-sheet and α-helix structures, respectively. Blue and orange arrows highlight the outer and inner membrane sides of the loop structures, respectively. Acidic and basic residues are indicated by blue and red letters, respectively; amino acids representing amido or hydroxyl group side-chains indicated by black letters; and hydrophobic amino acids shown as pale green. Sequences showing a trend from neutral to alkaliphilic (D + E) + (N + T + S) > (H + K + R) are indicated by blue boxes, and those showing a trend from neutral to alkaliphilic (D + E) + (N + T + S) < (H + K + R) indicated by green boxes.

**Figure 5:**
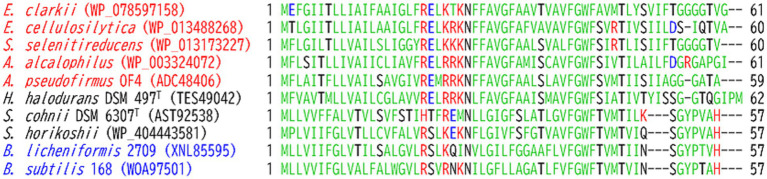
Amino acid sequence alignment of the small membrane-bound protein (DUF2759) equivalent to BpOF4_01690 (Takahashi et al., 2018) from *Evansella clarkii* DSM 8720^T^ and other alkaline and neutral hydrophilic *Bacillaceae*. Obligate and facultative alkaliphilic strains are indicated by red and black, respectively, and neutralophilic strains are shown by blue. Acidic and basic residues are indicated with blue and red letters, respectively; amino acids representing amido or hydroxyl group side-chains indicated by black letters; hydrophobic amino acids presented as pale green.

**Figure 6:**
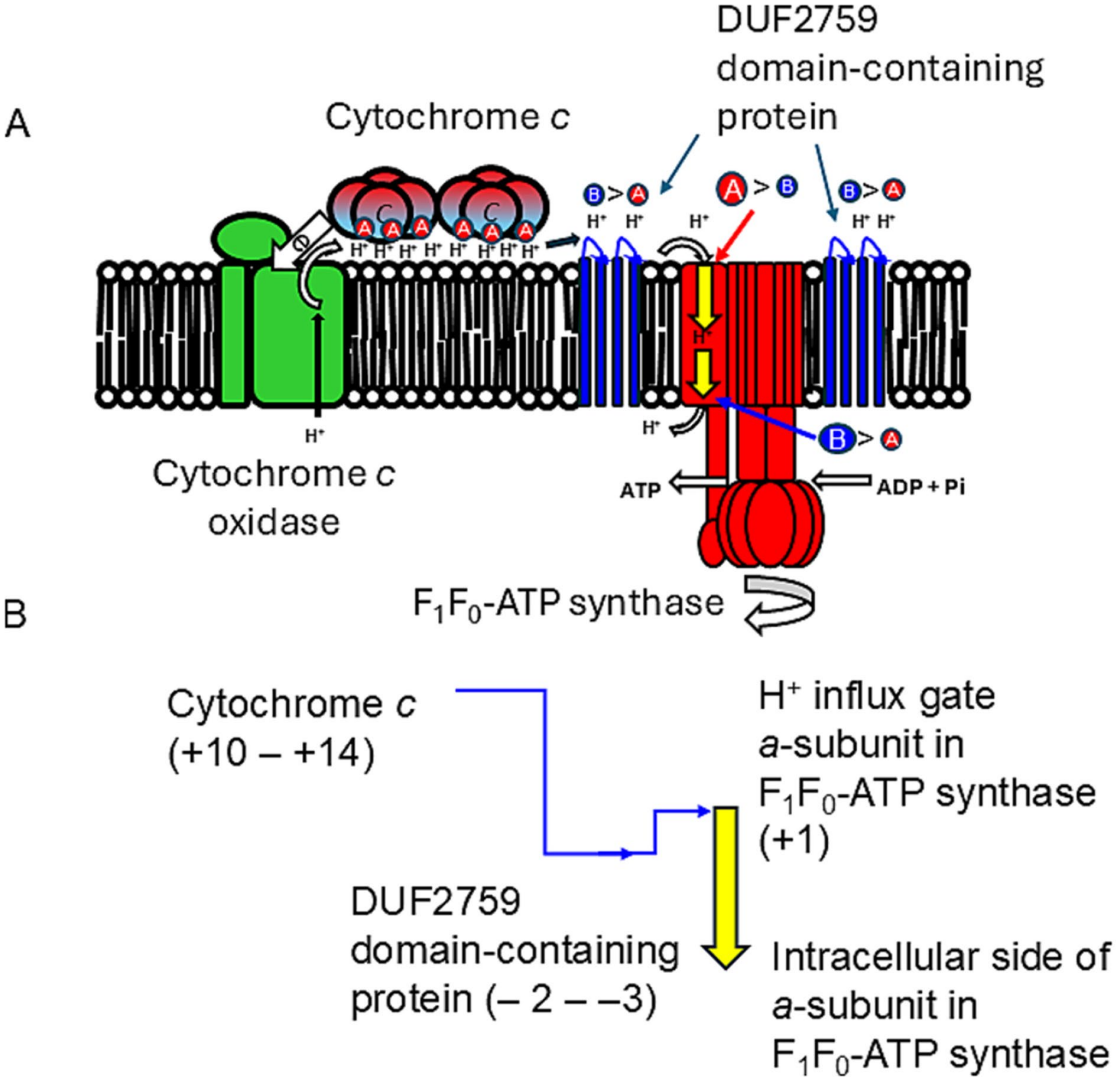
High efficiency in H^+^ localization on the outer surface of the membrane is achieved through a hydrogen-bonded network formed by the membrane-bound cytochrome c N-terminal segment (corresponding to Asn^4^-Asn^27^ in *E. clarkii* cytochrome c) in obligate alkaliphiles. **(A)** H^+^ capacitor formation and H^+^ transfer in the outer surface of membrane and F_1_F_0_-ATP synthase. The membrane-bound cytochrome *c* acts as an H^+^ capacitor due to its N-terminal segment. This segment is rich in acidic, amido-, and hydroxyl groups of amino acids, which, along with its tetrameric structure, play a crucial role in the accumulation and transfer of H^+^ on the outer membrane surface. H^+^ is transferred from the H^+^-capacitor by cytochrome *c* to F_1_F_0_-ATP synthase via a DUF2759 domain-containing protein (illustrated in blue). This protein features four or three basic amino acids and one acidic amino acid, which facilitate H^+^ recruitment near the H^+^ influx gate of the a-subunit of F_1_F_0_-ATP synthase. The disparity in the abundance of acidic (red A) versus basic (blue B) amino acids in the H^+^ carrier segment suggests its H^+^ release (refer to Table 2). The data indicate significant differences in H^+^ releasing and accepting capabilities between the outer membrane side (red arrow) and the intracellular side (blue arrow) of F_1_F_0_-ATP synthase in alkaliphiles. **(B)** Changes in amino acid composition in H^+^ transfer segments. This illustrates the changes in the abundance of acidic to basic amino acids in H^+^ carrier segments, highlighting the H^+^ transfer cascade from cytochrome *c* to F_1_F_0_-ATP synthase. It also presents a schematic representation of the high performance of F_1_F_0_-ATP synthase. Notably, a substantial gap exists between cytochrome *c* and the DUF2759 domain-containing protein that recruit H^+^. This larger gap is essential for overcoming the reversal difference between the DUF2759 domain-containing protein and the H^+^ influx gate of the a-subunit of F_1_F_0_-ATP synthase.

The increase in the abundance of acidic amino acids, paucity of basic amino acids, and increase in the amino acids contributing to H^+^ transfer via the hydrogen-bonding network (Marx et al., 1999; Marx, 2006) for cytochromes *c* observed in obligate alkaliphiles are higher than those in the other categorized members. Those differences are not as intense as those in the respiratory complexes. To draw the accumulated H^+^ from cytochrome *c* to the H^+^ influx gate in the *a*-subunit of F_1_F_0_-ATP synthase, a relative abundance of protonable basic amino acids should be present at the site. A multiple alignment was performed for the *a*-subunit of F_1_F_0_-ATP synthase because it serves as an H^+^ gate for the production of ATP (Vik et al., 2000). The most pronounced differences between the *a*-subunit of obligate alkaliphilic and the other categories of strains (facultative alkaliphiles and neutralophiles) are the N-terminal sequences that localize to the outer membrane surface (Figure 4). However, deprotonatable acidic amino acids at the N-terminal sequences that localize to the outer membrane surface in the *a*-subunit slightly outnumber basic ones. Therefore, H^+^ are likely transferred from cytochrome *c* to F_1_F_0_-ATP synthase via a small membrane-bound protein (BpOF4_01690) found in *A. pseudofirmus* OF4 (Figure 5; Takahashi et al., 2018). This protein exposes four or three basic amino acids and one acidic amino acid, may recruit H^+^ at the vicinity of the H^+^ influx gate of F_1_F_0_-ATP synthase. This protein has been reported to play a crucial role in oxidative phosphorylation (Takahashi et al., 2018). The difference in abundance of acidic and basic amino acids in an H^+^ carrier segment may indicate its ability to release or accept H^+^. Acidic *minus* basic amino acid residue numbers in the H^+^-transferring segments indicate a H^+^ releasing ability with a negative value, indicating H^+^-release or acceptability. The number of acidic *minus* basic amino acids in the amino acid sequence corresponding to the N-terminal domain of Asn_4_-Asn_37_ in cytochrome *c*, DUF2759 domain-containing protein (equivalent to BpOF4_01690), *a*-subunit of the H^+^ influx gate, and intracellular side of F_1_F_0_-ATP synthase in *E. clarkii* are +10, −2, +1, and −6, respectively (Table 2; Figure 6). Therefore, significant gaps could be present in the H^+^-releasing and -accepting abilities between the outer and intracellular membrane sides of F_1_F_0_-ATP synthase in alkaliphiles. Such acidic *minus* basic amino acid residue-related number gaps could also be observed between the N-terminal site of the cytochrome *c* and H^+^-recruiting small membrane-bound protein amino acid sequences. The above-described H^+^ might facilitate H^+^ transfer from the H^+^-capacitor in cytochrome *c* to the intracellular side of F_1_F_0_-ATP synthase. The higher abundance of protonatable amino acids in the low pH environment at the intracellular side of the F_1_F_0_-ATP synthase may be of some importance in driving H^+^ from the outer membrane surface to the intracellular space through F_1_F_0_-ATP synthase.

**Table 2:** Number of amino acids (acidic, basic and H^+^ transfer assist) that associated H^+^ transfer from cytochrome *c* to at the outer-surface membrane of obligate alkaliphilic *Evansella clarkii*, facultative alkaliphilic *Sutcliffiella cohnii*, and neutralophilic *Bacillus subtilis* and *Alkalihalophilus pseudofirmus*.

Proton-transferring segment	Acidic residues^a^	Basic residues^b^	Amido or hydroxyl amino acid^c^	Acidic *minus* basic amino acid^d^
***E. clarkii***				
Cytochrome *c* Asn^4^-Asn^37^	**10**	0	10	+10
DUF2759^e^	1	**3**	1	−2
F_1_F_0_-ATP synthase *a*-subunit extracellular side	**4**	3	7	+1
F_1_F_0_-ATP synthase *a*-subunit intracellular side	0	**6**	10	−6
***S. cohnii***				
Cytochrome *c* Gly^5^-Asp^24^	**5**	0	5	+5
DUF2759^d^	1	**2**	2	−1
F_1_F_0_-ATP synthase *a-*subunit extracellular side	**4**	3	4	+1
F_1_F_0_-ATP synthase *a-*subunit intracellular side	0	**5**	7	−5
***B. subtilis* 168**				
Cytochrome *c* Asn^4^-Glu^23^	**6**	3	5	3
DUF2759	0	**3**	2	−3
F_1_F_0_-ATP synthase *a*-subunit extracellular side	1	**4**	5	−3
F_1_F_0_-ATP synthase *a*-subunit intracellular side	0	**5**	10	−5
***A*. *pseudofirmus* OF4**				
Cytochrome *c* Gly^4^-Asn^38^	**14**	0	3	+14
DUF2759	1	**4**	1	−3
F_1_F_0_-ATP synthase *a*-subunit extracellular side	**5**	4	5	+1
F_1_F_0_-ATP synthase *a*-subunit intracellular side	0	**6**	10	−6

The bold numbers represent the number of acidic or basic amino acids that contribute to the proton affinity of each proton transfer segment.

a Acidic amino acid resides: D + E.

b Basic amino acid resides: H + K + R.

c Amido (N, Q) and hydroxyl (S, T) group side-chains possessing amino acid resides.

d Acidic minus basic amino acid residues.

e Small membrane bound protein equivalent to BpOF4_01690 (Takahashi et al., 2018).

Alkaliphilic *Bacillaceae* strains have been reported to harbor specific amino acid sequences in the *c*-subunit of F_1_F_0_-ATP synthase compared with those of neutralophilic strains (Liu et al., 2011). A multiple alignment was performed for the *c*-subunit of F_1_F_0_-ATP synthase in obligate and facultative alkaliphilic and neutralophilic *Bacillaceae* (Supplementary Figure S3). The outer membrane surface-exposed amino acid sequence of obligate alkaliphilic *E. clarkii* contains a higher frequency of amino acids that contribute to the hydrogen-bonding network and a lower frequency of basic amino acids compared with those of facultative alkaliphilic and neutralophilic sequences. Three Glu (E) residues were observed in neutrophilic *B. subtilis*, whereas in the three obligate alkaliphilic species, *E. clarkii*, *Salisediminibacterium selenitireducens*, and *E*. *cellulosilytica*, only one Glu (E) residue was observed among the 20 residues on the outer-surface side. These results suggest that the outer-surface side of the *c*-subunit residues in F_1_F_0_-ATP synthase is also designed to balance H^+^-acceptance and release.

## 7 Bioenergetic parameter and capacitance formation

From the amino acid alignment and characteristics of the amino acid sequence, we can predict the physiological role of *E. clarkii* cytochrome *c*-550. In addition, the measurements of bioenergetic parameters (cytochrome *c* content in the membrane, oxygen consumption rate during respiration, *ψ*, and ratio of H^+^ extrusion to oxygen consumption [H^+^/O]) will be necessary to refer to the outcome (ATP production rate). From the oxygen consumption rate and H^+^/O, we can predict the H^+^ extrusion frequency•min^−1^. By considering estimations of the H^+^ extrusion frequency•min^−1^ and ATP production•min^**−**1^, we can predict the actual potential for H^+^ extruded at the outer membrane for ATP production. Therefore, we could possibly verify the physiological role of cytochrome *c*-550 from *E. clarkii* and the value of H^+^ transfer by comparing bioenergetic parameters and the outcomes.

### 7.1 H^+^ translocation frequencies

The oxygen consumption rates of the obligate alkaliphile *E. clarkii* and the facultative alkaliphile *S*. *cohnii* are lower than that of the neutralophile *B. subtilis*. This difference may be attributed to the higher membrane electrical potential (Δ*ψ*) observed in alkaliphilic species. The H^+^-translocation by the respiratory complex is coupled to electron transfer to molecular oxygen. The translocation of positively charged protons (H^+^) from the intracellular to the extracellular side is impeded by this Δ*ψ*, which tends to attract cations back toward the cytoplasmic side of the membrane (Table 3).

**Table 3:** Summary of the bioenergetic parameters of obligate alkaliphilic *Evansella clarkii,* facultative alkaliphilic *Sutcliffiella cohnii* and neutralophilic *Bacillus subtilis* high aeration conditions*.

Bacterial strain	Cytochrome *c* content (nmol• mg^−1^)	Δ*ψ* (mV)	Respiratory rate (μmol O_2_ min^−1^•mg^−1^)	H^+^/O	Expected extruded H^+^ (min^−1^•mg^−1^)	Maximum ATP synthesis rate (nmol‧min^−1^ •mg^−1^)	Maximum ATP synthesis rate/ H^+^ (nmol/H^+^)
*E. clarkii* DSM8720^T^	0.36 ± 0.01	−192 ± 3	0.19 ± 0.04	2.2 ± 0.2 (6)^§^	0.84	26.2 ± 1.7	31.2 ± 2.0
*S. cohnii* YN-2000	0.62 ± 0.01	−173 ± 5	0.20 ± 0.03	2.6 ± 0.3 (6)	1.04	9.6 ± 0.9	9.23 ± 0.86
*B. subtilis* IAM 1026	0.21 ± 0.02	−121 ± 7	0.50 ± 0.06	4.9 ± 0.1 (6)	4.80	2.0 ± 0.6	0.42 ± 0.13

*These data are cited from Goto et al. (2016, 2022).

The cells were cultured in peptone and yeast extract -based PYA medium shaken at 120 rpm at 27 °C (Goto et al., 2016).

Alkaliphiles and *B. subtilis* are grown at pH 10 and 7, respectively.

^§^Numbers in parentheses are based on the theoretically extruded H^+^ in 1/2O_2_ consumption via the respiratory chain.

Additionally, the H^+^/O ratio—representing the number of protons extruded per oxygen molecule consumed—is lower in alkaliphiles than in *B. subtilis* (Table 3). One possible reason is the accumulation of H^+^ at the outer membrane surface, facilitated by cytochrome *c*, which may act as a limiting factor for efficient proton extrusion. This localized proton crowding could generate a back-pressure effect, impeding further H^+^ translocation by the respiratory chain. As a result, the rate of H^+^ export mediated by the respiratory chain in alkaliphilic strains is likely much lower than in *B. subtilis*.

It has been reported that both the membrane electrical potential (Δ*ψ*) and the transmembrane pH gradient (ΔpH) act to hinder proton translocation simultaneously in the respiratory chain, thereby limiting the effective generation of membrane potential (Gregory and Ferguson-Miller, 1989). This inhibitory effect is more pronounced in alkaliphiles than in neutralophiles. Nevertheless, once protons are successfully translocated across the membrane despite these barriers, they exhibit a remarkably high energetic potential. Consequently, the driving force per proton (Δp) for ATP synthesis via the F₁F₀-ATP synthase is substantially greater in alkaliphilic species (Table 3). However, the retention of protons at the outer membrane surface—mediated by Δ*ψ* and surface-associated cytochrome *c*—may impede their lateral transfer to ATP synthase. Therefore, enhancing proton transfer mechanisms at the membrane surface, potentially through strategically positioned amino acid residues that facilitate proton mobility, could be critical for optimizing ATP synthesis efficiency in alkaliphilic bacteria.

### 7.2 Transmembrane electrical potential

**Figure 7:**
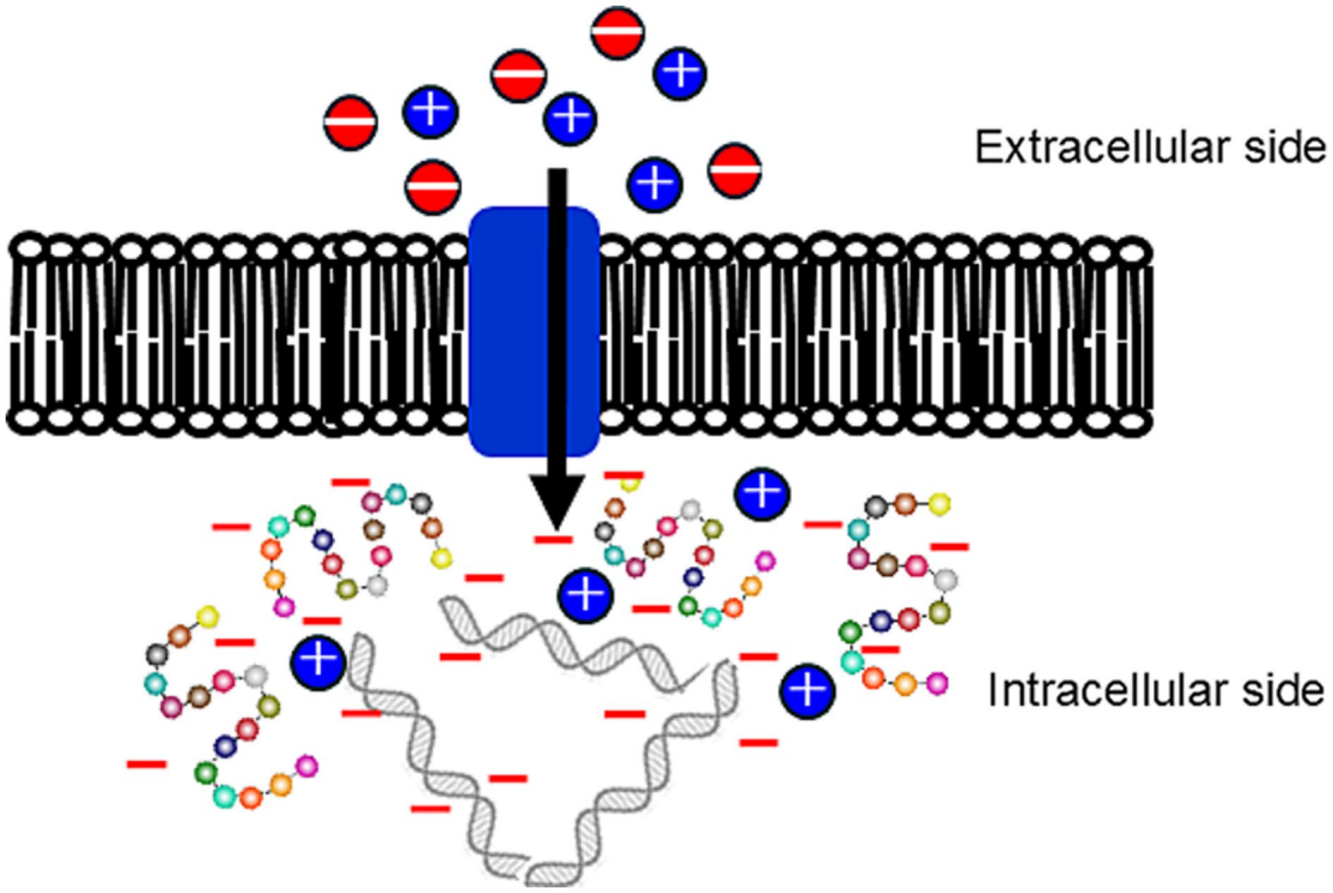
In alkaliphilic bacteria, the abundance of acidic proteins and nucleic acids in the intracellular space makes the intracellular side more likely to be negatively charged. This generates a strong membrane potential (Δ*ψ*), or high inward electrical potential for H^+^, between the intracellular space and the high-pH external environment. It is thought that the Donnan effect contributes to the formation of this potential. The Donnan potential is an electrical potential difference across a membrane caused by charged molecules (e.g., acidic proteins within cells) that cannot diffuse across a semi-permeable barrier (e.g., a cell membrane), resulting in a distribution bias of diffusible ions (e.g., Na^+^, Cl^−^).

**Figure 8:**
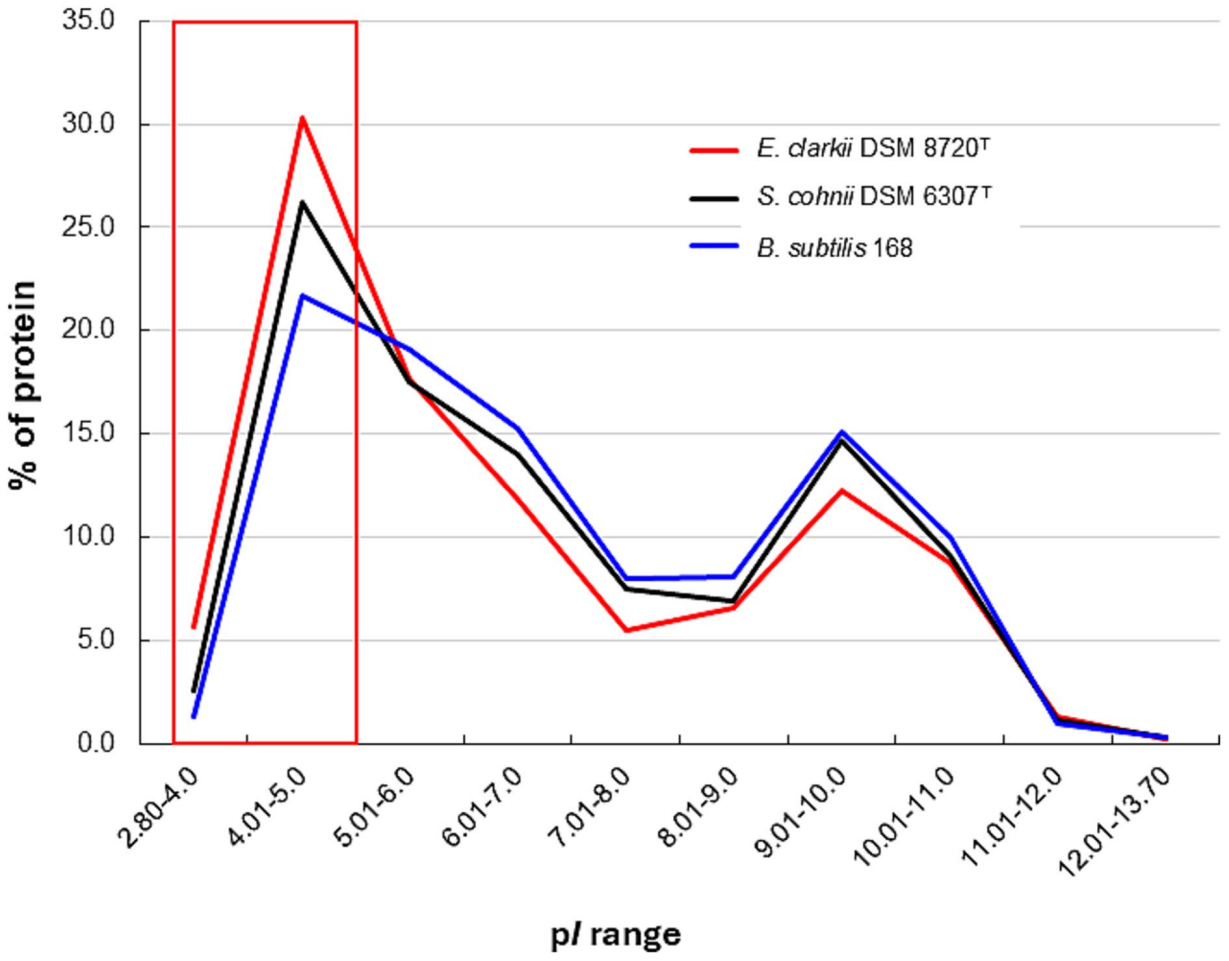
Protein isoelectric point (p*I*) distribution in obligate alkaliphilic *Evansella clarkii* DSM 8720^T^, facultative alkaliphilic *Sutcliffiella cohnii* DSM 6306^T^ and neutralophilic *Bacillus subtilis* 168. The red line indicates *E. clarkii* DSM 8720^T^, black line indicates *S*. *cohnii* DSM 6306^T^, and blue line indicates *B. subtilis* 168. The red boxes indicate that the p*I* of proteins from obligate alkaliphilic *E. clarkii* DSM 8720^T^ is biased toward the low-pH side compared with that of facultative alkaliphilic *S*. *cohnii* DSM 6306^T^ and neutralophilic *B. subtilis* 168. The p*I* values of each genome were calculated using the Sequence Manipulation Suite server (Stothard, 2000).

Δ*ψ* is the voltage difference across a biological membrane, which is usually between the inside and outside of a cell or organelle is a key component of the electrochemical gradient that drives many cellular processes. In bacteria, the inside of the membrane (cytoplasmic side) is generally negatively charged, whereas the outside (external environment side) is positively charged. This potential difference is mainly caused by differences in ion concentration and membrane permeability. For measurements of the bacterial Δ*ψ*, cationic probes permeable to the membrane have often been used and according to the distribution across the membrane Δ*ψ* (*V*_m_), can be calculated via the Nernst equation:


$$V_{m}=\frac{\mathrm{RT}}{\mathrm{zF}}\;\text{In}(\frac{C_{\text{out}}}{C_{\text{in}}})$$

where *V*_m_ is the equilibrium potential of the ion (=Δ*ψ*), z represents the number of moles of electrons transferred in the electrochemical reaction and *C_out_* and *C_in_* are the ion concentrations outside and inside the membrane, respectively. One of the main sources of the bacterial Δ*ψ* is the Donnan potential (Donnan, 1924). This occurs when negatively charged molecules that are impermeable to the membrane are present on the intracellular side of the membrane (Figure 7). Intracellular DNA was shown to be naturally charged. In addition, negatively charged proteins corresponding to the intracellular pH are thought to contribute to the Donnan potential. Acidic proteins with a low isoelectric point (p*I*) will exhibit the intracellular pH of alkaliphilic bacteria (pH 8.1–8.4 when the extracellular pH is approximately 10). Considering the Donnan potential that contributes to the Δ*ψ*, the intrinsic non-energized Δ*ψ* (Δ*ψ*_NE_) can be assumed to be attributable to the initial ion balance across the membrane and existence of negatively charged intracellular molecules. However, H^+^ are translocated through respiratory activity of the energized Δψ (Δ*ψ*_E_), which affects ATP production via F_1_F_0_-ATP synthase. Intracellular DNA and acidic proteins contribute to increases in the Donnan potential, which subsequently contributes to the Δ*ψ*_NE_. Analysis of the predicted protein p*I* derived from whole genome analyses indicates that the obligate alkaliphile, *E. clarkii*, has a higher preference for acidic amino acids than the facultative alkaliphile, *S*. *cohnii*, and neutralophilic *B. subtilis* do (Figure 8). Lebre and Cowan (2020) also reported the preference of acidic proteins (p*I* 4.01–5) in alkaliphiles compared with that in neutralophiles, and acidophiles. The results of comparative gene expressions that correspond to culture conditions show that obligatory alkaliphilic *E. clarkii* has a predisposition to exhibit a higher Δ*ψ*_NE_, which is attributed by the Donnan potential, than that of *S*. *cohnii* and *B. subtilis*. A negative ion capacity was estimated in *E. clarkii* and *S*. *cohnii* grown at pH 10 and *B. subtilis* grown at pH 7 (Goto et al., 2016). In addition, the Donnan potential can be predicted by the negative ion capacity of intracellular constituents. The capacity in two alkaliphilic strains increased as the studied pH increased in the range of pH 6–8 and remained at high values in the range of pH 8–10, whereas the values hardly changed at pH 6–10 in *B. subtilis*. As the Δψ_NE_ attracts H^+^ to the intracellular site, it hinders the transport of H^+^ via the respiratory chain. However, once translocated to the outer membrane surface, H^+^ will have a higher potential for Δp than in the case of a moderate Δ*ψ*_NE_. To overcome the difficulty of H^+^ translocation, cytochrome *c* and its moieties of respiratory complexes exhibit a lower redox potential compared with that in neutralophiles to produce a larger gap between the electron donor (cytochrome *c*) and acceptor (cytochrome *a*) in complex IV (Lewis et al., 1981; Orii et al., 1991).

Thus, the produced congenial high Δψ_NE_ can lead to a high Δψ_E_. The effect of the ψ_Ε_ retention of extruded H^+^ via the respiratory chain has been reported previously (Yoshimune et al., 2010). The rate of H^+^ extrusion during respiratory activity in *E. clarkii* is slower than that in *B. subtilis*. This suggests that H^+^ extruded through respiration in *E. clarkii* are retained at the outer membrane surface. However, the retained H^+^ are rapidly released into the bulk phase after the introduction of valinomycin, which disrupts the Δψ_Ε_. However, the retardation of H^+^ at the outer membrane surface was enhanced after the introduction of monensin, which acts as a Na^+^/H^+^ exchanger. This suggests that H^+^ accumulating at the outer membrane surface were exchanged for intercellular Na^+^.

### 7.3 Efficiency of ATP production

To estimate the ATP production efficiency of each strain (*E. clarkii*, *S*. *cohnii*, and *B. subtilis*), the rate of maximum ATP synthesis per H^+^ translocated by the respiratory chain was estimated (Table 3). This experimental system used endogenous substrates. Less than 20% of ATP can be assumed to be derived from substrate-level phosphorylation (Russell and Cook, 1995). The maximum ATP synthesis efficiency per H^+^ extrusion rate of *E. clarkii* was approximately 3.4 times higher than that of *S*. *cohnii*, whereas it was approximately 130-fold higher than that of *B. subtilis*. This high efficiency cannot be explained by the high Δψ_Ε_ alone; the H^+^-capacitor function is likely responsible for this, which involves the retention of accumulated cytochrome *c* and H^+^ on the outer membrane surface and their transfer to the F_1_F_0_-ATP synthase. This configuration creates an additional imbalance across the membrane in the electrochemical *Η*^+^ potential that exists along the outer membrane surface for driving F_1_F_0_-ATP synthase. Thus, alkaliphiles strategically employ even more effective bioenergetic mechanics to reverse the harsh alkaline environment.

## 8 Conclusion and perspective

Obligate alkaliphilic *E. clarkii* and facultative alkaliphilic *S*. *cohnii* exhibit a phylogenetic position near that of neutralophilic *B. subtilis*; however, these species are able to grow vigorously under harsh environments at pH 10 in which ordinary neutralophiles are unable to grow. This review focused on the H^+^-transferable amino acids at the N-terminal side of membrane-bound cytochromes *c* that can effectively accumulate H^+^ on the outer membrane surface, which forms a H^+^-capacitor. Thus, the produced H^+^-capacitor would bring an additional unbalanced vertical force to drive F_1_F_0_-ATP synthase via H^+^ concentrations and electrical charges across the membrane. Furthermore, the maximal driving performance of proton translocation and ATP synthesis in *E. clarkii* is likely achieved through a steep proton affinity gradient between the efficient H^+^ transfer across the outer membrane surface and the H^+^ entry site of the F₁F₀-ATP synthase on the cytoplasmic side. The proton transfer mechanism is governed by the differential abundance of acidic and basic amino acid residues within key protein segments. Specifically, the net number of acidic minus basic residues in the amino acid sequences corresponding to the N-terminal domain of cytochrome *c*, the DUF2759 domain-containing protein, the a-subunit of the H^+^ influx gate, and the intracellular side of the F₁F₀-ATP synthase are +10, −2, +1, and −6, respectively. This pronounced cascade of proton affinity differences exemplifies a unique bioenergetic adaptation characteristic of alkaliphilic bacteria. Taken together, these findings suggest that the H^+^-capacitor mechanism involving cytochrome *c* plays a contributory role in driving ATP synthesis. In the future, verifying the physiological role of the N-terminal sequence of cytochrome *c*-550 using various mutant proteins, and clarifying the relationship between the amino acid sequence and H^+^ retention ability would be crucial.

Formation of a high Δ*ψ*_E_ is indispensable for the high performance of an H^+^-capacitor and physiological functioning in alkaliphilic *Bacillaceae*. The total bacterial transmembrane potential consists of charge separation driven by heterologous permeable ions across the membrane, impermeable substances across the membrane (Donnan potential), and chemical modifications to the membrane surface (e.g., membrane lipid head group) (Benarroch and Asally, 2020). Among them, charge separation driven by the existence of intracellular, impermeable substances with strong negative charges are intrinsically advantageous to produce large amounts of ATP in the obligate alkaliphilic *E. clarkii*. The experimentally confirmed intracellular negative charge (Goto et al., 2016) may be largely attributable to the presence of negatively charged proteins (Figure 6). However, these proteins and their biochemical processes have yet to be identified. These negative charges use the intracellular pH of alkaliphiles, which are approximately one unit higher (i.e., ca. pH 8) than that in neutralophilic bacterial strains. The intracellular negative ion capacity decreases under low aeration conditions in both *E. clarkii* and *S*. *cohnii* (Goto et al., 2016). Thus, these alkaliphiles can regulate the expression of negatively charged substances by sensing external environmental conditions. Identification of substances contributing to the intracellular negative ion capacity and expression of associated regulatory mechanisms should be clarified in future studies.

Alkaliphilic bacteria have a lower respiratory rate than that of neutrophiles. This is not only due to the lack of H^+^, but also to the inhibition of transfer through the Δψ that attribute to an intrinsic, large, intracellularly negative ion capacity. Alkaliphilic bacteria strategically produce an even higher membrane potential than neutralophiles do because they must maintain the function of an even more complicated solute transport system. Under high pH conditions, alkaliphilic bacteria have thus adopted the H^+^-capacitor system. This mechanism increases the driving force of F_1_F_0_-ATP synthase by allowing H^+^ to accumulate on the outer membrane surface, thereby driving F_1_F_0_-ATP synthase more efficiently. In addition, as the positive charge of H^+^ accumulates on the outer membrane surface, this likely creates an attractive force between the positive and negative charges present, which is equivalent to the electrostatic attraction observed in an actual electrical capacitor (opposite charge), providing the driving force for F_1_F_0_-ATP synthase propulsion. The presence of a factor equivalent to the electrostatic attraction should be verified in future studies.
