# Supplementary material for: H+-capacitor and ATP production in obligate alkaliphilic Bacillaceae: insights into cytochrome c and H+ transport mechanisms
Source: Front Microbiol. 2025 Sep 10;16:1637315. doi: 10.3389/fmicb.2025.1637315 (PMC12457358; doi:10.3389/fmicb.2025.1637315)
Supplement: Supplementary file 1 [file Supplementary_file_1.docx]

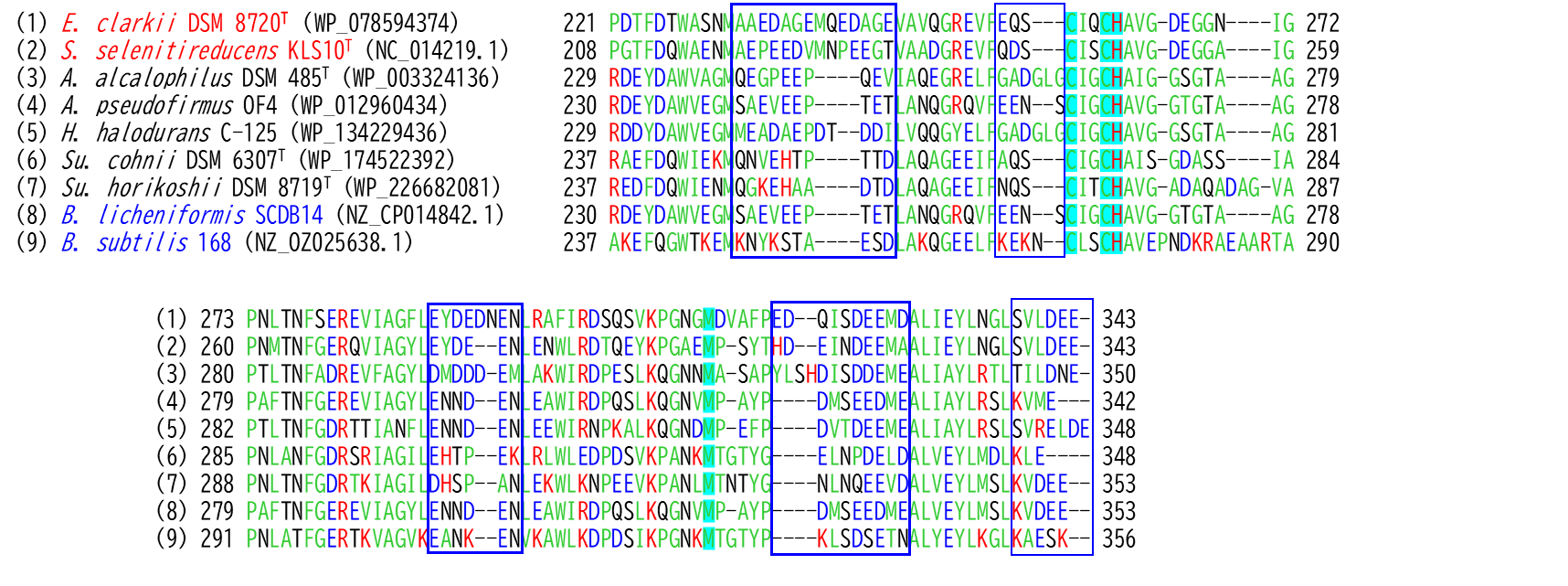
Supplementary Figure 1ǀ Amino acid sequence alignment of the cytochrome c segment of cytochrome caa_3_ subunit II from Evancella clarkii and other alkaliphilic and neutralophilic Bacillaceae. Obligate and facultative alkaliphilic strains are indicated by red and black text, respectively, and neutralophilic strains shown by blue text. Amino acids representing the heme-binding site (C) and axical ligands (H and M) are indicated by a light-blue marker. Acidic (D, E) and basic (H, K, and R) residues are indicated by blue and red letters, respectively, amino acids representing amido (N, Q) or hydroxyl (S, T) group side-chains are indicated by black letters; hydrophobic amino acids are shown as pale green. Sequences showing a trend from neutral to alkaliphilic (D+E) + (N+Q+T+S) ˃ (H+K+R) are indicated by blue boxes.


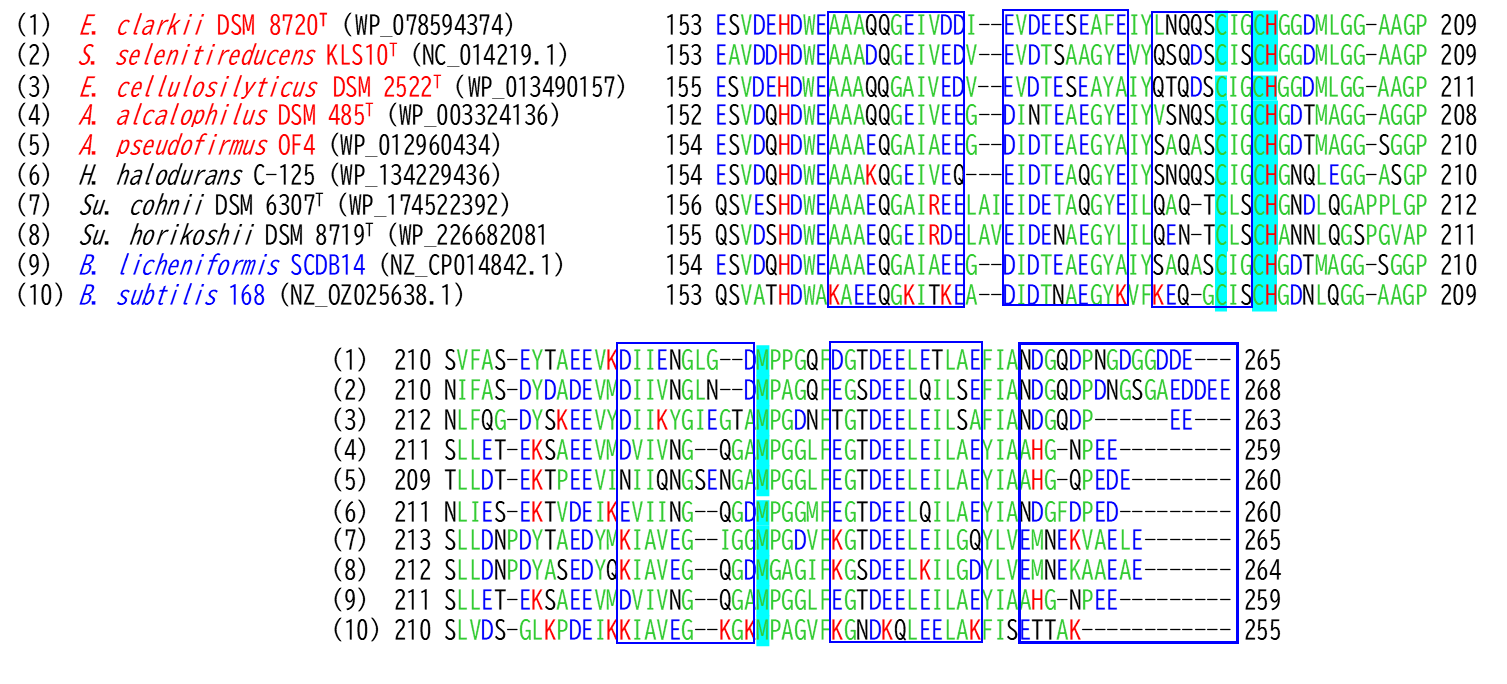


**Supplementary Figure 2**ǀ Amino acid sequence alignment of the cytochrome *c* segments of cytochrome *bc*_1_ from *Evancella* *clarkii* and other alkaliphilic and neutralophilic *Bacillaceae*. Obligate and facultative alkaliphilic strains are indicated by red and black, respectively, and neutralophilic strains shown by blue text. Amino acids representing the heme-binding site (C) and axical ligands (H and M) are indicated byS a light-blue marker. Acidic (D, E) and basic (H, K and R) residues are indicated by blue and red letters, respectively; amino acids representing amido (N, Q) or hydroxyl (S, T) group side chains amino acids are indicated by black letters; and hydrophobic amino acids are shown as pale green. Sequences showing a trend from neutral to alkaliphilic (D+E) + (N+Q+T+S) ˃ (H+K+R) are indicated by blue boxes.


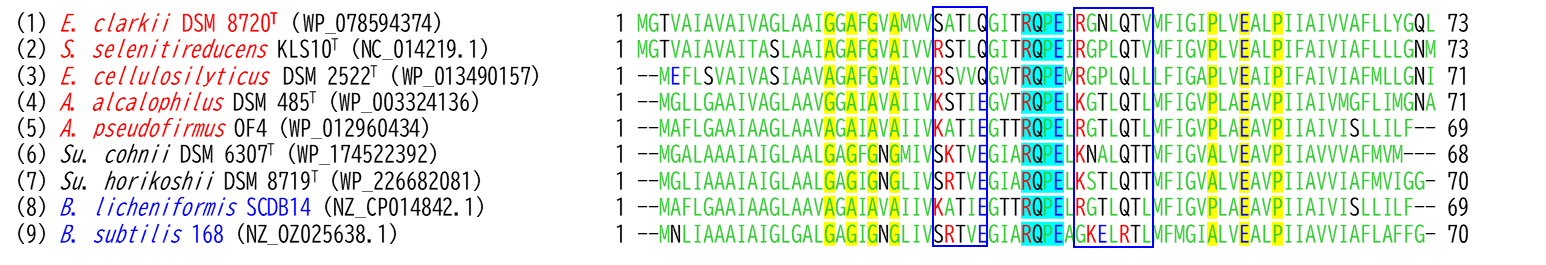


**Supplementary Figure 3**ǀ Amino acid sequence alignment of the *c*-subunit of F_1_F_0_-ATP synthase from *Evansella* *clarkii* and other alkaliphilic and neutralophilic *Bacillaceae*. Obligate and facultative alkaliphilic strains are indicated by red and black text, respectively, and neutralophilic strains shown by blue text. The loop is indicated by a light-blue marker. Corresponding positions of the GXGXGXG and AXXEXXP motifs observed in several non-alkaliphilic strains are indicated by yellow markers (Hicks et al., 2010). Acidic (D, E) and basic (H, K and R) residues are indicated by blue and red letters, respectively, amino acids representing amido (N, Q) or hydroxyl (S, T) side- chains are indicated by black letters; and hydrophobic amino acids are shown as pale green. Sequences showing a trend from neutral to alkaliphilic (D+E) + (N+Q+T+S) ˃ (H+K+R) are indicated by blue boxes.
